# Squamate scavenging services: Heath goannas (*Varanus rosenbergi*) support carcass removal and may suppress agriculturally damaging blowflies

**DOI:** 10.1002/ece3.11535

**Published:** 2024-06-25

**Authors:** Tom J. M. Jameson, Gregory R. Johnston, Max Barr, Derek Sandow, Jason J. Head, Edgar C. Turner

**Affiliations:** ^1^ Department of Zoology and University Museum of Zoology University of Cambridge Cambridge UK; ^2^ College of Science & Engineering Flinders University Adelaide South Australia Australia; ^3^ South Australian Museum Adelaide South Australia Australia; ^4^ Northern and Yorke Landscape Board Minlaton South Australia Australia; ^5^ Northern and Yorke Landscape Board Clare South Australia Australia

**Keywords:** ecosystem function, ecosystem services, introduced species, reptiles, rewilding, scavenging

## Abstract

Human‐induced environmental change has caused widespread loss of species that support important functions for ecosystems and society. For example, vertebrate scavengers contribute to the functional health of ecosystems and provide services to agricultural landscapes by removing carcasses and associated pests. Widespread extirpation of native Australian mammals since the arrival of Europeans in Australia has removed many scavenging species from landscapes, while scavenging mammals such as European red foxes (*Vulpes vulpes*) have been introduced. In much of Australia, squamate reptiles are the largest native terrestrial scavengers remaining, where large native mammals are extinct and conservation management is being undertaken to remove invasive mammals. The contribution of reptiles to scavenging functions is not well understood. In this study, we investigated the ecosystem functions provided by large reptiles as scavengers to better understand how populations can be managed to support ecosystem services. We investigated the ecosystem services provided by vertebrate scavengers in Australian coastal mallee ecosystems, focusing on the heath goanna (*Varanus rosenbergi*), the only extant native terrestrial scavenger in the region. We carried out exclosure experiments, isolating the scavenging activity of different taxonomic groups to quantify the contribution of different taxa to scavenging services, specifically the removal of rat carcasses, and its impact on the occurrence of agriculturally damaging blowflies. We compared areas with different native and invasive scavenger communities to investigate the impact of invasive species removal and native species abundance on scavenging services. Our results indicated that vertebrate scavenging significantly contributes to carcass removal and limitation of necrophagous fly breeding in carcasses and that levels of removal are higher in areas associated with high densities of heath goannas and low densities of invasive mammals. Therefore, augmentation of heath goanna populations represents a promising management strategy to restore and maximize scavenging ecosystem services.

## Graphical abstract

Vertebrate scavenging in Australia significantly contributes to carcass removal and suppresses the breeding of agriculturally harmful blowflies. Levels of carcass removal are higher in areas associated with higher densities of native reptiles and lower densities of invasive mammals. Therefore, augmentation of reptile populations and control of invasive mammals represents a promising management strategy to restore and maximize scavenging ecosystem services and reduce the incidence of blowfly induced diseases.

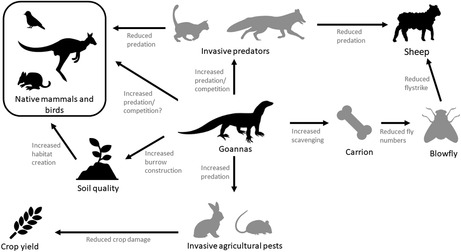

## 1 INTRODUCTION

Ecosystems across the world are missing species as a result of human actions (Ceballos et al., 2015). These absences reduce the diversity of functions that an ecosystem can provide (Weiskopf et al., 2020). Ecosystem functions such as scavenging provide important services for society such as limiting the spread of disease by removing carcasses in which pathogens breed (Vicente & Vercauteren, 2019) and maintaining soil fertility through nutrient cycling (Barton et al., 2013; Benbow et al., 2019; Cunningham et al., 2018). Identification and remediation of missing or diminished ecosystem functions are central to rewilding conservation approaches which aim to return functions to ecosystems through the reintroduction of locally extinct species or their analogues (Barnosky et al., 2017; Carver et al., 2021; Lorimer et al., 2015; Svenning et al., 2016).

Carcass removal by scavenging is an important ecosystem service in Australia. Removal of carcasses can prevent the attraction of unwanted animals to an area (e.g., dingos, *Canis dingo*; black rats, *Rattus rattus*; red foxes, *Vulpes vulpes*; and sheep blowflies, *Lucilia cuprina* [Diptera: Calliphorida]), which can benefit local human communities by limiting the spread of diseases (e.g., rabies, leptospirosis, and flystrike) and the damage that unwanted pest species may cause to livestock, crops, property, and native ecosystems (O'Bryan et al., 2018; Ogada et al., 2012; Peisley et al., 2017). Red foxes are a major agricultural pest in southern Australia, contributing up to 30% of lamb mortality on sheep farms in the southern Yorke Peninsula (SYP) (Johnston & Menz, 2019; Sharp, 2018).

Primary blowflies, such as *Lucilia cuprina*, *Calliphora nociva* (Diptera: Calliphorida), and *Calliphora albifrontalis* (Diptera: Calliphorida) are other key agricultural pest, causing flystrike (cutaneous myiasis) in sheep; a condition whereby blowfly maggots burrow into the flesh of sheep, causing painful wounds that often become infected and acting as vectors for other pathogens and parasites (Bansode et al., 2017; Godoy et al., 1997; Smith, 2021). Flystrike contributes to sheep deaths, loss of body condition and associated value, and reduced breeding success (Horton et al., 2018; Smith & Wall, 1997). Through these effects, flystrike costs the Australian sheep farming industry an estimated $280 million annually (Smith, 2021). Carcasses are a major source of flystrike guild flies, with over 70% of flies emerging from carcasses in sheep pastures in New Zealand found to be members of the flystrike guild (Heath & Appleton, 1999). The removal of carcasses from the vicinity of sheep may therefore be a potential approach for the control of flystrike (Heath & Appleton, 1999; Smith & Wall, 1997; Wall et al., 1995).

In Australia, scavenging is primarily undertaken by corvids (Bragato et al., 2022; Peisley et al., 2017; Read & Wilson, 2004), native marsupials (Cunningham et al., 2018; Vandersteen et al., 2023), introduced and naturalized mammalian carnivores (Newsome & Spencer, 2022; Spencer & Newsome, 2021; Vandersteen et al., 2023; Wirsing & Newsome, 2021), and specialist invertebrates (Read & Wilson, 2004). The relative contribution of different groups to scavenging is highly dependent on season and habitat type (Bragato et al., 2022; Read & Wilson, 2004; Vandersteen et al., 2023) and exclusion of mesoscavengers by larger species (Cunningham et al., 2018; Vandersteen et al., 2023). No studies have formally investigated the contribution of squamate reptiles to scavenging ecosystem functions in Australia, with most vertebrate studies focused on mammals and birds (Barton et al., 2013). However, in some parts of Australia squamate reptiles are the largest native terrestrial scavengers remaining, especially in areas where large marsupial scavengers (e.g., Tasmanian devils, *Sarcophilus harrisii*) and large naturalized scavengers (e.g., Dingos) are extinct or absent. Predation by introduced European red foxes and cats (*Felis catus*) has been a major cause of native wildlife loss and ecological degradation in Australia (Woinarski et al., 2019). While foxes and cats act as scavengers themselves, they have also reduced the abundance of native scavengers. In areas where conservation management is being undertaken to remove invasive foxes and cats, large squamate reptiles such as goannas (genus *Varanus*) are the largest remaining scavengers in the landscape. As such, it is important to understand the contribution of large squamate reptiles to scavenging ecosystem functions.

**FIGURE 1:**
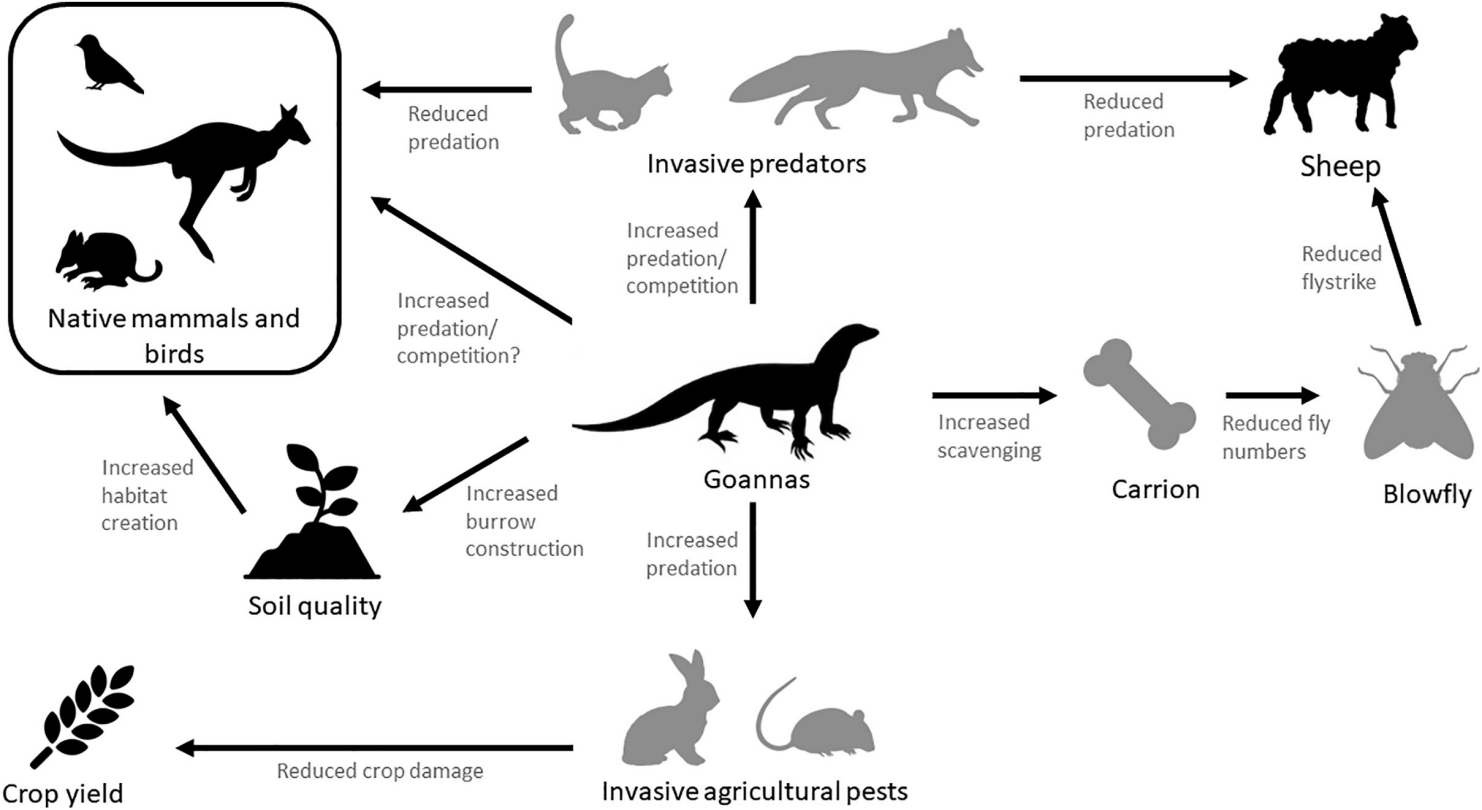
Schematic of potential ecological interactions between heath goannas (*Varanus rosenbergi*) and other species and ecological processes within Australian coastal mallee landscapes. Arrows indicate the ecological effects of an increased goanna population. Species/processes in black are predicted to increase in response to an increased goanna population, whilst species/processes in gray are predicted to decrease.

Previous studies of scavenging ecosystem functions have identified heath goannas (*Varanus rosenbergi*) as potentially important scavengers in southern Australia (O'Brien et al., 2007), while rewilding projects have also noted that knowledge gaps exist with regards to what function the species provides and how it should be managed (Johnston & Menz, 2019) (Figure 1). The heath goanna is a mid‐sized (50 cm snout—vent) terrestrial predator and scavenger, endemic to the heathlands of southern Australian, and listed as endangered under the South Australian National Parks & Wildlife Act (1972) and vulnerable under the New South Wales National Parks & Wildlife Act (1974). Like many other species of *Varanus*, the heath goanna may play an important role in its ecosystem as a predator (de Miranda, 2017), scavenger (Dalhuijsen et al., 2014; Kulabtong & Mahaprom, 2015; Read & Wilson, 2004; Shine et al., 1998), nutrient linker (Boshoff et al., 1994; Mukherjee & Sen Sarkar, 2013; Stewart et al., 1997; Sutherland, 2011), and soil engineer (Doody et al., 2021). In addition, the heath goanna may be an effective biological control agent of agricultural pests and invasive species, like other species of *Varanus* (Cota, 2008; Greer, 1989; Mirtschin, 1982; Robinson et al., 1985; Uchida, 1966).

In this study, we investigated the ecosystem services that heath goannas provide as scavengers in Australian coastal mallee ecosystems (semi‐arid landscapes dominated by low‐growing eucalyptus scrub). We carried out exclosure experiments in areas with scavenger communities made up of differing proportions of heath goannas and invasive mammals to assess the contribution of different species to scavenging services, specifically the removal of carcasses and the limitation of necrophagous fly breeding. We addressed the following questions: (1) Which vertebrate species contribute to scavenging on carcasses in Australian coastal mallee landscapes? (2) How long do different scavenging vertebrates take to find carcasses? (3) Do vertebrate scavengers significantly reduce carcass mass, relative to invertebrate scavengers in isolation? (4) Do vertebrate scavengers significantly reduce the number of necrophagous flies breeding in carcasses? (5) Do higher goanna densities lead to a greater reduction of carcass mass and necrophagous fly breeding in carcasses? We predicted that heath goannas would be key scavengers in Australian coastal mallee ecosystems, and that areas with higher goanna densities would benefit from a greater availability of ecosystem services, such as more rapid carcass removal and more intense suppression of necrophagous flies, relative to areas with lower densities.

## 2 METHODS

### 2.1 Study area

**FIGURE 2:**
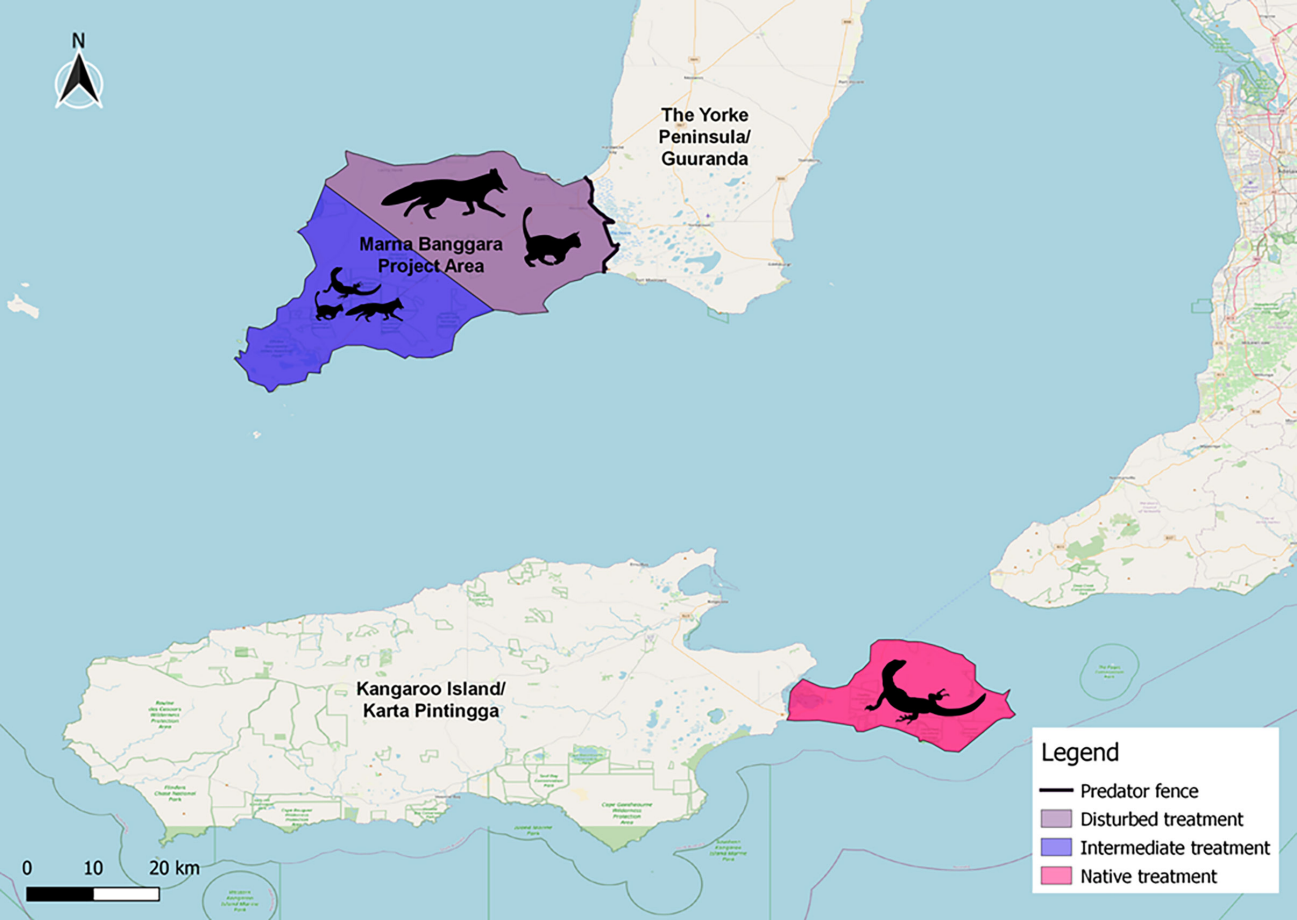
The study area with each scavenger community treatment highlighted. Silhouettes show a simplified representation of the major differences in each treatment's scavenger community.

We conducted our study at the Marna Banggara Project on Guuranda, the SYP, and on the Dudley Peninsula of Karta Pintingga/ Kangaroo Island (KI), South Australia (Figure 2). Marna Banggara is a rewilding project taking place on the SYP, which is restoring the landscape by removing foxes and cats and reintroducing native species to reinstate ecosystem functions such as scavenging (Johnston & Menz, 2019). KI is Australia's third‐largest island and lies less than 45 km south of the SYP across the Investigator Strait. The two regions were connected by a land bridge between 8800 and 9900 years ago and the island contains similar coastal mallee habitat to the SYP (Hope et al., 1977). However, unlike the SYP, KI has never had a population of European red foxes. Although the Island does have a population of feral cats, an intense eradication program is underway, with a particular focus on the Dudley Peninsula on the east of the island (Hohnen et al., 2020, 2022). Therefore, despite the underlying similarity of habitat and climate, the scavenger communities are very different between the SYP and Dudley Peninsula. On the SYP, foxes and cats are present in large numbers and heath goannas are absent or occur in low densities, whereas on the Dudley Peninsula foxes are absent, cats occur at reduced densities and heath goannas occur at very high densities (Goanna Watch, 2013; Rismiller et al., 2010). As such, KI is an excellent control area for the SYP, allowing an assessment of the ecosystem functions provided by heath goannas in the absence of foxes, as well as providing an insight into the potential level of scavenging activity by heath goannas on the SYP once foxes are removed.

The study landscapes are a patchwork of cultivated and pastoral farmland alongside areas of native vegetation. Native vegetation areas on the SYP were characterized by Johnston and Menz (2019) and are similar to those found on KI. The majority of native vegetation patches on the SYP are found within protected areas, including the National Park (Dhilba Guuranda‐Innes National Park), Conservation Parks (Warrenben, Leven Beach, Thinda, Point Davenport), and privately owned Heritage Vegetation blocks. Much of the south of the Dudley Peninsula consists of native vegetation communities that encompass several public and privately owned conservation parks.

We carried out exclosure experiments at 18 sites across the SYP and 9 sites on KI (Figure S1). We placed half of the experimental sites on the SYP in the north‐eastern half of the landscape (Figure 2), further from the National Park and closer to the predator exclusion fence (where invasive mammal baiting is less intense and has only been present since 2012), in sites >5 km from any goanna sightings since 2014 as recorded by long‐term monitoring efforts (Jameson et al., in press). We placed the other half of the SYP sites in the south‐western half of the study landscape (Figure 2), closer to the National Park, further away from the predator exclusion fence (where invasive mammal baiting is more intense and has been present since 2006), and within 1 km of a location where there has been a heath goanna sighting since 2014 (Jameson et al., in press). We placed the KI sites on the Dudley Peninsula, where no foxes were present, an intense cat eradication program is ongoing, and goanna densities were high. This gave us three distinct landscape treatments that differed in terms of the scavenger community, allowing us to assess whether the make‐up of the scavenger community alters carcass removal and necrophagous fly breeding. The sites in the northeast of the SYP represented a more disturbed scavenger community (disturbed treatment) with many invasive scavengers and no native goannas. In contrast, the sites on the Dudley Peninsula represented a more natural scavenger community (native treatment), with no foxes, fewer cats, and large numbers of goannas. The sites in the southwest of the SYP represented a scavenger community intermediate between the disturbed and native treatment, with intermediate levels of invasive mammal control and goanna densities (intermediate treatment).

We conducted scavenging experiments at 18 sites on the SYP between March and April 2022, we repeated experiments at these sites and at 9 additional sites on the Dudley Peninsula between October and December 2022. Early and late summer were chosen as these are the points in the year at which heath goannas undertake the most active foraging (Rismiller et al., 2010), allowing us to investigate the maximum scavenging contribution of the species. We located all sites in areas of native vegetation of the same habitat type as far as possible, which consisted of mallee bushland dominated by kingscote mallee and coastal white mallee (*Eucalyptus rugosa* – *E. diversifolia*). We placed all sites within protected areas, both public land and privately owned Heritage Agreement vegetation plots.

We split the nine sites in each treatment into three clusters of three sites, each cluster within separate patches of native vegetation (Figure S1). We spread site clusters roughly evenly across the landscape to capture spatial variability across treatments, with sites set up in clusters for logistical reasons, allowing all sites in the same cluster to be serviced in a single day. Within clusters, we placed the three sites >1 km from each other to create spatially independent sites, ensuring that scavenging species would need to find each site independently.

### 2.2 Feeding stations

**FIGURE 3:**
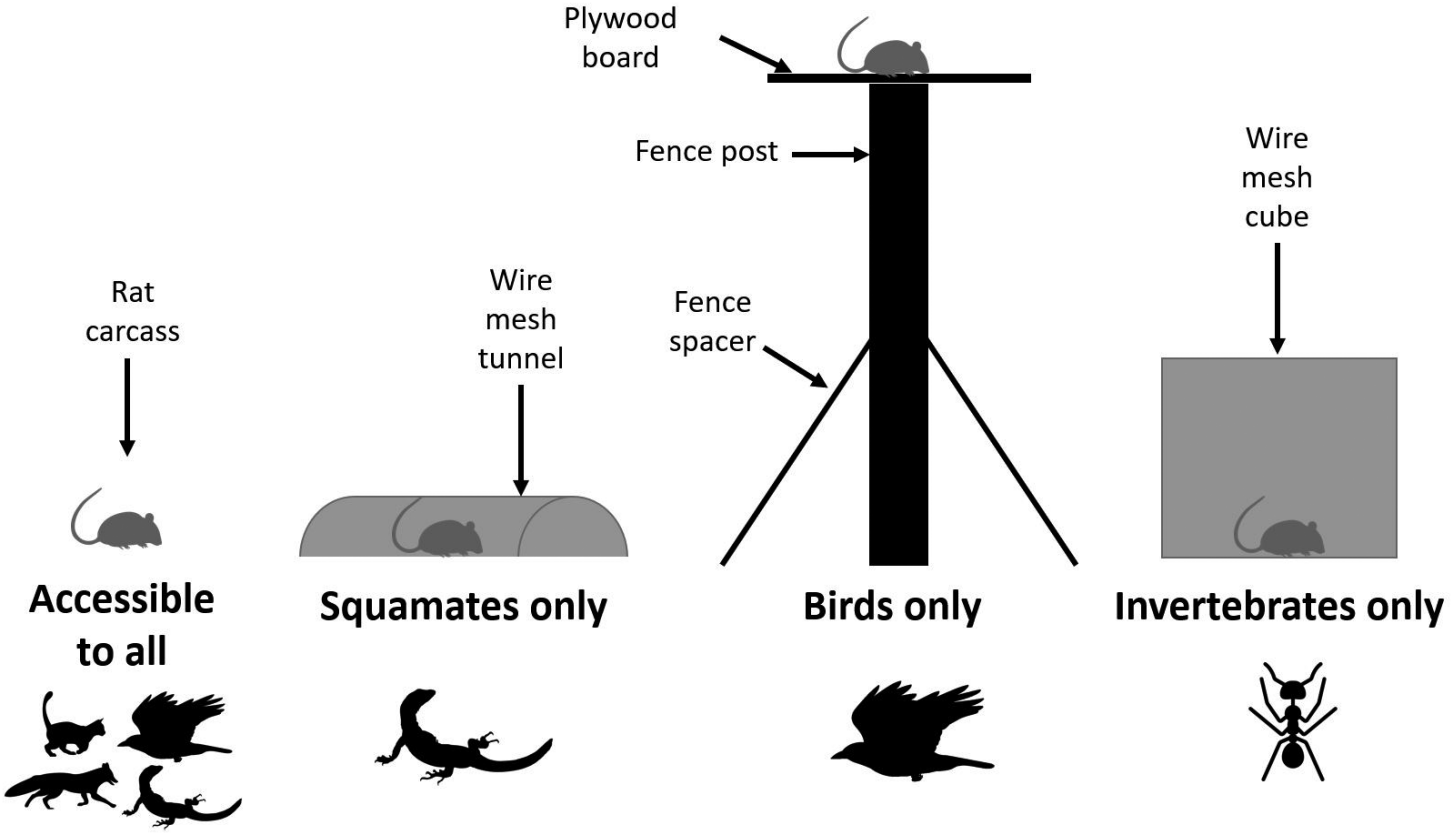
Schematic of feeding stations. Silhouettes show a simplified representation of taxa that can access each station.

At each experimental site, we setup four feeding stations (total = 180 feeding stations), each baited with a single petfood‐grade whole rat carcass (mean ± standard deviation = 139 g ± 30 g), purchased from a local pet food supplier (Peninsula Nursery & Pets, Kadina, South Australia). We weighed carcasses to the nearest gram prior to placement. We attached carcasses to feeding stations with cable ties to prevent animals from removing whole carcasses. Adapting the methods of Peisley et al. (2017), we setup feeding stations as follows (Figure 3):

1. Accessible to all – This station imitated natural conditions, located on the ground, and completely open and accessible to terrestrial animals, birds, and invertebrates. Carcasses were cable‐tied to ground‐level vegetation.
2. Accessible only to squamates – This station excluded birds and terrestrial mammals. It consisted of a 50 cm long wire‐mesh tunnel, built by bending a 50 cm × 30 cm wire‐mesh panel into a U‐shape. The tunnel was secured to the soil using U‐shaped metal planting stakes, three equally spaced along each side of the tunnel. Each carcass was then cable‐tied to wire‐mesh at the center of the tunnel.
3. Accessible only to birds – This station excluded terrestrial scavengers following the design of Peisley et al. (2017). It consisted of a 2 m high raised platform. These stations were constructed by attaching a 50 cm × 50 cm plywood board to the top of a 2 m tall fence post. Stations were installed in the field by sinking the pole as deep into the ground as possible (<5–20 cm dependent on local soil depth). Once sunk into the ground, four fence spacers were then attached to the base of the tower at right angles to act as “legs” to keep the tower stable.
4. Accessible only to invertebrates – This station excluded all vertebrate scavengers. The station consisted of a 50 cm × 50 cm × 50 cm cubic cage on the ground built from wire‐mesh panels attached together using aviary clips. Cages were secured to the ground using U‐shaped planting stakes, one in each corner of the base of the cube. Carcasses were attached to the bottom center of the cubes with cable ties.

Following the logic of Peisley et al. (2017), excluding non‐squamate scavengers (station 2) meant that any loss in carcass weight was due to squamates and invertebrates only, and allowed an assessment of the role of squamates in carcass breakdown. Similarly, excluding terrestrial scavengers (station 3) allowed an assessment of the role of birds in carcass breakdown. We compared stations 1, 2, and 3 with station 4 to determine the role of each vertebrate group in carcass breakdown, relative to that by invertebrates alone.

### 2.3 Recording scavenger species

We monitored stations 1–3 with Reconyx Hyperfire HC600 camera traps to record vertebrate visitors. Camera trap setup followed the methodology of Peisley et al. (2017). We attached all camera traps to star droppers hammered into the ground. Where possible, we positioned the cameras 2–4 m from the feeding station, so that the entire station was in view and facing south to reduce distorted images due to sun glare. The cameras recorded the time, date, and ambient temperature when each image was taken. We programmed cameras at high sensitivity and to take a burst of three images each time they were triggered to increase the likelihood of identifying photographed animals. We recorded these three photos as one “event” and used them to record information about the identity of animals accessing the carcasses. We set cameras to not have any quiet period between triggers, to ensure that all visits to carcasses were recorded. We used the “walk‐test” function to ensure that animals visiting carcasses would trigger the cameras.

We recorded all vertebrate scavengers present at feeding stations based on camera trap images. We recorded species presence as a visit if a species was present but not actively feeding, but as a scavenging event if a species was actively feeding on a carcass. We treated visits and scavenging events by the same species concurrently as distinct events if >30 min had elapsed between visits.

### 2.4 Carcass removal and necrophagous fly analysis

Following site setup, carcass exposure ran for 5 days. We chose 5 days as an appropriate exposure period because primary Australian blowflies (*Lucilia cuprina*, *Calliphora nociva*, *Calliphora albifrontalis*) leave carcasses to pupate in the surrounding soil ~5 days after eggs are laid (116–161 h) (Bansode et al., 2017; Monzu, 1978). This allowed carcasses to be collected prior to the onset of larvae leaving carcasses, allowing the maximum number of larvae within carcasses to be counted (Sawyer et al., 2022). Furthermore, pilot studies carried out in February of 2022 found that after 5 days, carcasses were too decomposed or desiccated to support further necrophagous fly breeding. As such, this exposure period ensured that the maximum potential number of necrophagous flies breeding in a carcass could be quantified.

Following the 5‐day exposure period, we collected and re‐weighed carcasses to the nearest gram to calculate the mass change in each carcass during the exposure period. Before re‐weighing, we removed any invertebrates (larvae and adults) in the carcass to ensure that an accurate measure of carcass mass could be made and to collect invertebrates for analysis. We removed invertebrates by placing carcasses in sealed plastic bags and systematically massaging the carcass by applying pressure from the center outward anteriorly and posteriorly for a standard 2 min. This pushed invertebrates out of the carcass into the plastic bag, via orifices and holes ripped in the carcass by scavengers. We chose 2 min as a standard amount of time, as pilot studies found that the majority of invertebrates were removed within this timeframe. We transferred collected invertebrates into 50 mL sample tubes and preserved them using 70% denatured ethanol solution.

We counted the number of necrophagous fly larvae (maggots) present in each carcass, as a proxy for the effect of scavenging activity on primary blowfly species. Previous studies have found that the majority of necrophagous flies emerging from small mammal carcasses in Australasian sheep pastures are members of the flystrike guild (Heath & Appleton, 1999), but we acknowledge that this may not be the case in this study.

Although not all maggots were extracted from carcasses using this method, we expected the relative number of removed maggots to be comparable between carcasses. To test this assumption, we dissected 18 rats following maggot extraction to count the total number of maggots left within each carcass. A simple linear regression showed that the number of maggots removed (*s*) was a significant predictor of the total number of maggots (*t*) in a carcass (*F* = 167.36, df = 1,16, *p* = .00), whereby *t* = 76.266 + 1.144 s. A mean of 75% of total maggots were removed by our method (standard deviation 16%). Further analyses investigated the relative difference in maggot counts between different carcasses, therefore, we did not adjust maggot counts to reflect the total number of maggots per carcass, as removed maggots were a reliable estimate of the total number per carcass.

### 2.5 Data analysis

We performed all statistical analyses in R version 3.5.1 (R Core Team, 2019). We carried out data exploration following (Zuur et al., 2010). We tested for normality of data using Shaprio‐Wilk tests and homoscedasticity using Levene tests. We investigated the distribution of dependent variables using *fitdistrplus* (Delignette‐Muller & Dutang, 2014). We fitted general linear mixed models (GLMMs) using *glmmTMB* (Magnusson et al., 2019). We validated models by plotting residuals in *DHARMa* (Hartig, 2022). We determined the significance of independent covariables to each model by comparing fitted models with null models using likelihood ratio tests (LRTs).

### 2.6 Time taken to find carcasses

We quantified the time taken by a species to find a carcass, by calculating the time elapsed between a feeding station being setup and the time at which species presence triggered the camera. We used a one‐way ANOVA to test if there was a significant difference in the mean time taken for different taxonomic groups (Birds, Squamates, and Mammals) to find carcasses and carried out post hoc analysis using a Tukey's honest significant difference test.

### 2.7 Effect of vertebrate scavengers on carcass removal and necrophagous fly breeding

We pooled data from all feeding station types to analyze the difference in carcass mass change and in maggot count per carcass between carcasses where vertebrate scavenging had occurred compared with where it had not. To do this, we constructed GLMMs of both mass change and maggot count per carcass, including vertebrate scavenging on carcasses (Yes or No) as a fixed effect and site and season (March–April and October–December) as a random effects in both cases. The inclusion of site and season as random effects accounted for the potential effect of weather and temperature on scavenger activity and potential differences in maggot counts between carcasses driven by the response of maggot developmental rate to different temperatures (Bansode, et al., 2017; Sawyer et al., 2022). We also included the starting mass of carcasses as a random effect in the mass change models to account for the potential of larger carcasses preferentially attracting scavengers. We applied a square‐root transformation to the mass change data to normalize the distribution, this allowed us to fit the mass change models to a Gaussian distribution. The maggot count data had a negative binomial distribution, we therefore fitted maggot count models to this distribution with the logarithmically transformed starting mass of carcasses as an offset variable, this accounted for the expected greater number of maggots in larger carcasses. We also applied zero‐inflated terms to vertebrate scavenging events to account for the high number of zeros in the data where whole carcasses were eaten by vertebrate scavengers. For all models, we inspected residual plots to confirm that model assumptions of normality and homoscedasticity were met. We tested the significance of independent covariables to each model by comparing fitted models with null models using LRTs.

### 2.8 Effect of scavenging communities and taxa on carcass removal and necrophagous fly breeding

We constructed GLMMs to compare changes in carcass mass and maggot count per carcass between different kinds of feeding stations and different scavenger community treatments. For both mass change and maggot count models, we included feeding station type (accessible to all, squamates only, birds only, invertebrates only), scavenger community (disturbed, intermediate, native), and interaction of these two effects as fixed effects, and site and season (March–April and October–December) as random effects. We also included starting mass of carcasses as a random effect in the mass change models. We applied a square‐root transformation to the mass change data to normalize the distribution. This allowed us to fit the mass change models to a Gaussian distribution. The maggot count data had a negative binomial distribution, we therefore fitted maggot count models to this distribution, with the logarithmically transformed starting mass of carcasses as an offset variable. For all models, we inspected residual plots to confirm that model assumptions of normality and homoscedasticity were met. We tested the significance of independent covariables to each model by comparing fitted models with null models using LRTs.

Where LRTs found feeding station type to be significant, we reran GLMMs and LRTs with only vertebrate exposed feeding stations (accessible to all, squamates only, and birds only), to investigate differences between different vertebrate scavenging groups. Where LRTs found scavenger community treatment to be significant, we reran GLMMs and LRTs with only SYP scavenger community treatments (intermediate and disturbed) to directly investigate the impact of conservation interventions removing foxes and cats from the SYP on scavenging services.

## 3 RESULTS

### 3.1 Scavenger species in Australian coastal mallee

**FIGURE 4:**
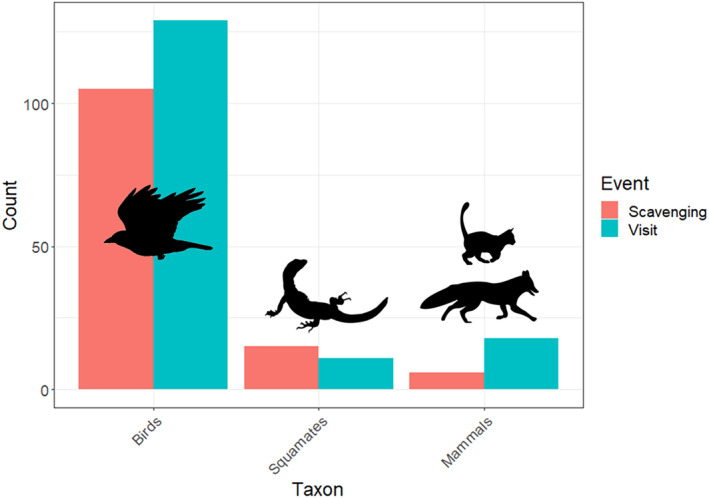
Visits and scavenging events to/on carcasses at feeding stations by vertebrates in Australian coastal mallee, based on data collated from all feeding stations that were accessible to vertebrate scavengers (accessible to all, squamates only, birds only – *N* = 135). Silhouettes show representations of each taxon.

A total of 61 of the 135 (45.19%) feeding stations exposed to vertebrate scavengers were scavenged on by vertebrates. Eight scavenging species were observed scavenging on carcasses and an additional two species were observed visiting carcasses (Figure 4). The little raven (*Corvus mellori*) was by far the most common species to visit and scavenge on carcasses (101 visits and 93 scavenging events), other avian scavengers included the gray currawong (*Strepera versicolor*) (22 visits and 5 scavenging events), Australian raven (*Corvus coronoides*) (5 visits and 1 scavenging event), tawny frogmouth (*Podargus strigoides*) (2 scavenging events), and gray butcherbird (*Cracticus torquatus*) (2 visits). Among squamate scavengers, heath goannas undertook a large amount of scavenging (14 visits and 7 scavenging events) with shinglebacks (*Tiliqua rugosa*) visiting carcasses on 4 occasions and scavenging on a carcass once. Among invasive mammals, cats, and foxes were also common visitors and scavengers at feeding stations (cats: 16 visits, 2 scavenging events; foxes: 4 visits, 2 scavenging events).

### 3.2 Time taken to find carcasses

**FIGURE 5:**
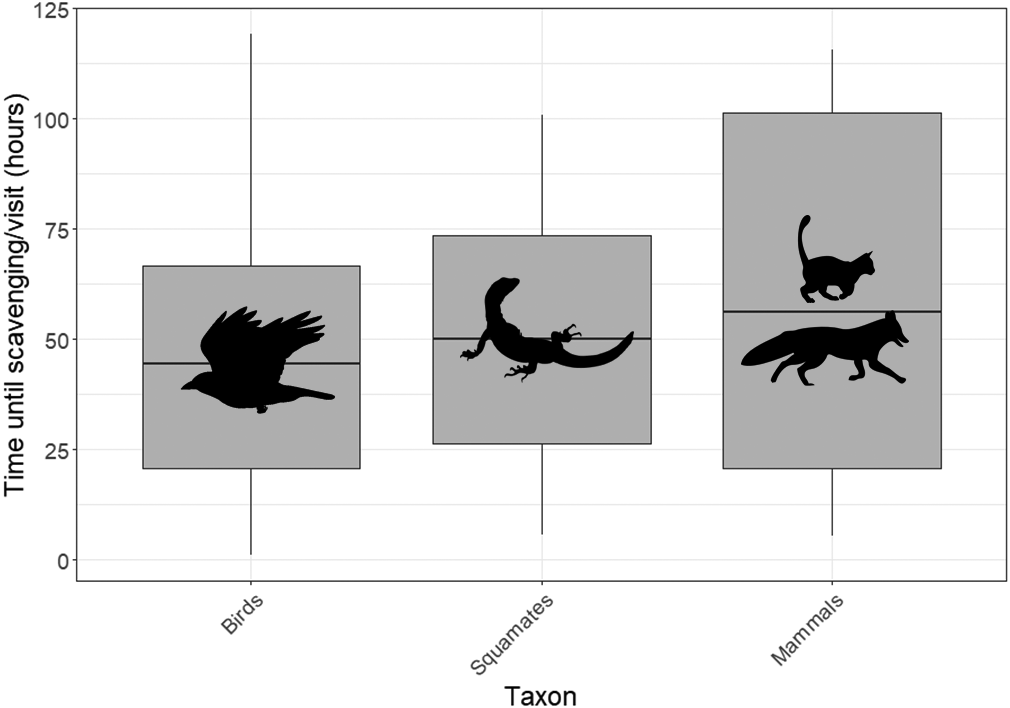
Time from site setup until presence at feeding stations by vertebrates in Australian coastal mallee, based on data collated from all feeding stations that were accessible to vertebrate scavengers (accessible to all, squamates only, birds only – *N* = 135), showing median (horizontal line), upper and lower quartiles (box boundaries), range of maximum and minimum values (whiskers), and potential outliers (>1.5 times the interquartile range – points). Silhouettes show representations of each taxon.

Time taken by scavengers to find carcasses varied between taxa (Figure 5). Birds were the first group to visit and scavenge on carcasses, taking a mean time of 28.51 h to visit and scavenge on a carcass, and a minimum of 1 h. Of bird species, gray currawongs were on average the earliest visitors to carcasses, taking a mean of 19.91 h. Squamates were the next fastest group to find carcasses (mean 41.22 h, minimum 5.53 h). Among squamates, heath goannas were on average the earliest visitors to carcasses, taking a mean of 39.76 h. Invasive mammalian scavengers took the longest of any group to find carcasses (mean 43.46 h, minimum 5.32 h). Among mammals, foxes were on average the earliest visitors to carcasses, taking a mean of 38.44 h.

The mean time taken by scavengers to find carcasses differed significantly between taxa (*F* = 4.63, df = 2283, *p* = .01). Birds found carcasses significantly earlier than mammals (*p* = .02), but there was no significant difference in the mean time taken to find a carcass between birds and squamates (*p* = .31) and squamates and mammals (*p* = .55).

### 3.3 Effect of vertebrate scavengers on carcass removal and necrophagous fly breeding

**FIGURE 6:**
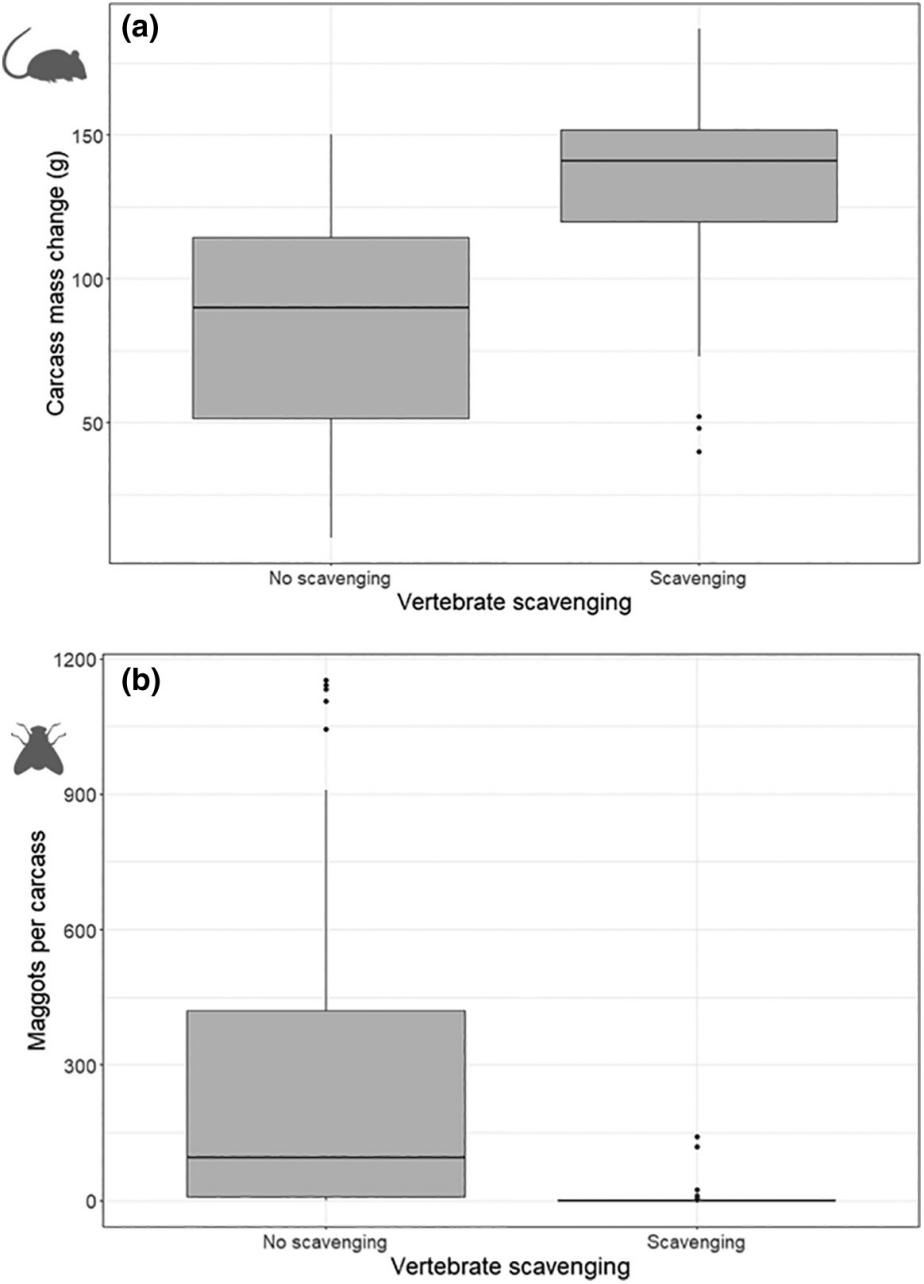
The effect of vertebrate scavenging on carcasses for data collated from all feeding stations (*N* = 180). (a) Carcass mass change (g) after a 5‐day exposure period and (b) the number of maggots per carcass after a 5‐day exposure period. Median (horizontal line), upper and lower quartiles (box boundaries), range of maximum and minimum values (whiskers), and potential outliers (>1.5 times the interquartile range – points).

Carcasses scavenged on by vertebrates lost significantly more mass than those not scavenged on by vertebrates (Figure 6a) (LRT = 96.66, *p* = .00), with vertebrate scavengers removing a median 141 g of carcass, relative to a median of 90 g in the absence of vertebrate scavenging. There were also significantly fewer maggots per carcass in carcasses scavenged on by vertebrates compared with those that were not (Figure 6b) (LRT = 8.11, *p* = .00), a median of 0 compared with 92.

### 3.4 Effect of scavenging communities and taxa on carcass removal and necrophagous fly breeding

**FIGURE 7:**
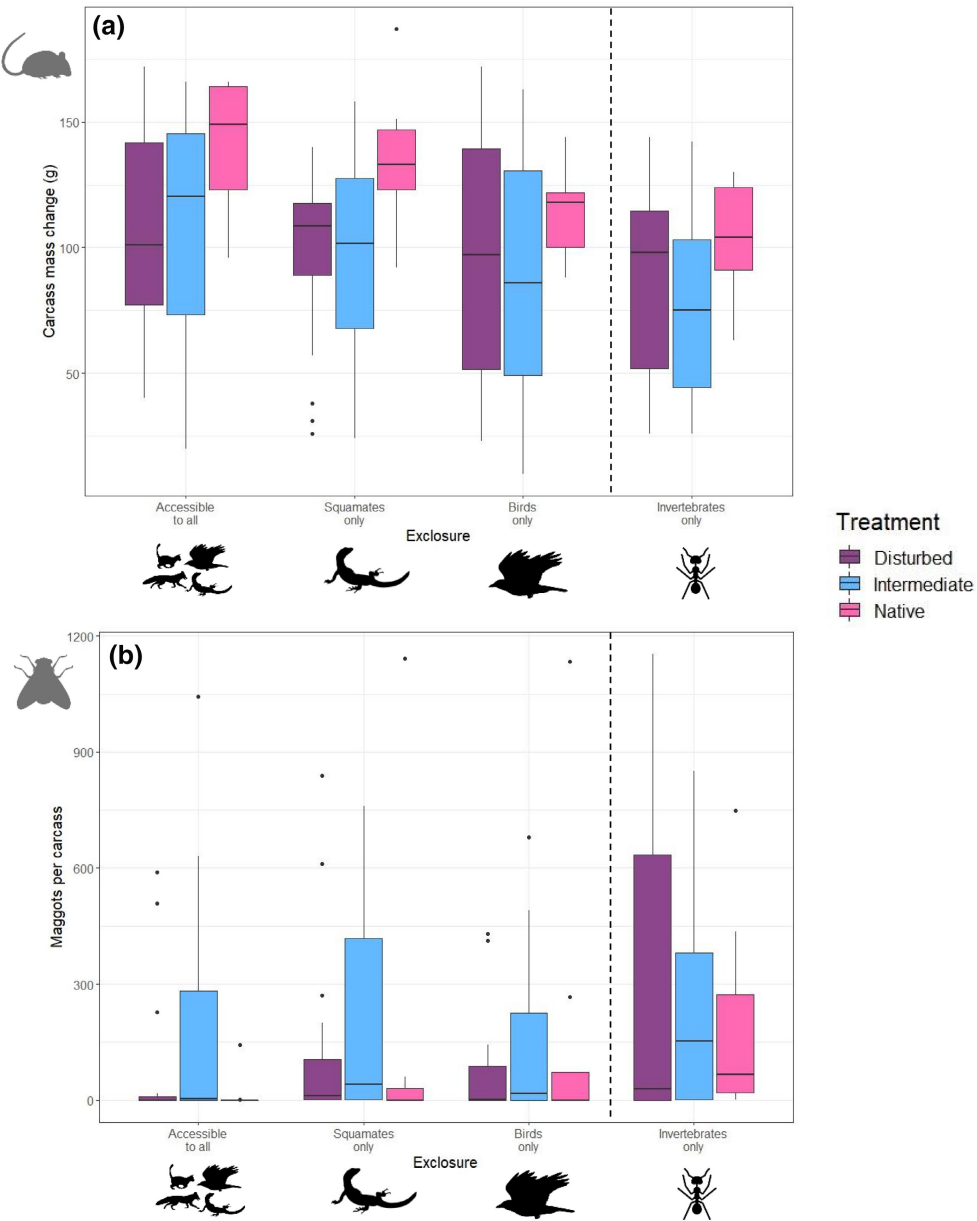
The effect of feeding station (*N* = 45 per station) and scavenger community treatment (*N* = 72 disturbed and intermediate treatment, *N* = 36 native treatment) on (a) the carcass mass change (g) after a 5‐day exposure period, and (b) the number of maggots per carcass after a 5‐day exposure period. Median (horizontal line), upper and lower quartiles (box boundaries), range of maximum and minimum values (whiskers), and potential outliers (>1.5 times the interquartile range – points).

Carcass mass change differed significantly between scavenger community treatments (LRT = 10.19, *p* = .01) and station type (LRT = 16.01, *p* = .00), but there was no significant interaction between these variables (LRT = 1.36, *p* = .71) (Figure 7a). Carcass mass change did not differ significantly between different vertebrate exposed feeding stations (LRT = 4.34, *p* = .11) or within SYP scavenger community treatments (LRT = 0.09, *p* = .76). A median of 123 g of carcass was removed in the native treatment relative to 99 g in other treatments. Vertebrate‐accessible stations had a median 115 g of carcass removed, relative to a median of 96 g at invertebrate‐only stations.

Station type had a significant effect on maggot density per carcass (LRT = 17.93, *p* = .00), but scavenger community treatment did not (LRT = 2.60, *p* = .27), nor was there an interaction between these two terms (LRT = 6.81, *p* = .34) (Figure 7b). Maggot density per carcass did not differ significantly between different vertebrate exposed feeding stations (LRT = 4.50, *p* = .11). Vertebrate‐accessible station carcasses had a median of 3 maggots compared with 91 in invertebrate‐only stations.

We found very high variance in carcass mass change and maggot density per carcass in the intermediate scavenger community treatments (Figure 7), particularly in carcasses only exposed to vertebrate scavengers.

## 4 DISCUSSION

### 4.1 Scavenger species in Australian coastal mallee

Vertebrate scavengers visited roughly 40% of available carcasses, consistent with previous studies (45.19% in this study, 41.67% in Peisley et al., 2017). Birds were the most common scavengers of carcasses, consistent with previous studies of Australian scavenging systems (Bragato et al., 2022; O'Brien et al., 2007; Peisley et al., 2017; Read & Wilson, 2004). Heath goannas were the second most common scavenger of carcasses. This is consistent with other scavenging studies that have occurred within the range of the heath goanna (O'Brien et al., 2007), but contrasts with studies elsewhere in Australia which have recorded minimal scavenging activity by squamate reptiles even during summer months (e.g. Newsome & Spencer, 2022; Read & Wilson, 2004; Vandersteen et al., 2023). This suggests that heath goannas play a particularly important role as scavengers on the south coast of Australia relative to squamates elsewhere on the continent.

Birds were the first scavengers to visit carcasses in our study, as has been found elsewhere in Australia (O'Brien et al., 2007; Peisley et al., 2017; Read & Wilson, 2004). The rapid discovery of carcasses by birds relative to other taxa may be attributable to flight facilitating faster movement from a higher vantage point than terrestrial species, increasing the probability of birds discovering carcasses relative to non‐volant taxa. Scavenging birds found carcasses significantly faster than invasive mammals, but there was no difference in time to find carcasses between birds and squamates, and squamates and invasive mammalian scavengers. Overall, there was a lot of overlap in the time taken for different scavenging groups to find and feed on carcasses. This suggests some redundancy between different scavenging groups in terms of the stage of decomposition at which they interact with carcasses. As such, different scavenging groups have similar scavenging effects upon carcasses, altering carcass interaction with the environment during decomposition in similar ways.

The greater activity of squamate scavengers in this study relative to other studies of scavenger ecosystem functions in Australia could reflect differences in carrion carcass sizes. Previous studies have used large mammal carcasses (typically kangaroos) (e.g. Newsome & Spencer, 2022; Read & Wilson, 2004; Vandersteen et al., 2023), whereas in this study we make use of smaller carcasses (rats). The use of relatively small carcasses allowed us to carry out experiments across many sites, providing us with large sample sizes and allowing us to account for spatial variability within landscape treatments. Furthermore, the use of small carcasses allowed us to differentially exclude different taxonomic groups of scavengers, which would have been more difficult or impossible with larger carcasses. However, data on scavenging activity at small carcasses may not be directly transferable to scavenging on larger carcasses. Previous studies have found that scavenger diversity at carcasses increases significantly in response to carcass size and that large carcasses typically attract large mammalian scavenger and obligate avian scavengers, while small carcasses are typically dominated by mesoscavengers (Moleón et al., 2015; Sebastián‐González et al., 2019; Turner et al., 2017). Our results are consistent with these findings, whereby vertebrate scavengers feeding on the small carcasses we used were exclusively mesoscavengers, with low diversity of scavengers at each carcass as most carcasses were consumed by one individual in a single visit. As such, we hypothesize that scavenging activity within Australian coastal mallee ecosystems may differ on larger carcasses, where we would expect to see the presence of larger scavengers such as wedge‐tailed eagles (*Aquila audax*). However, the only other scavenging study carried out in the range of the heath goanna, which used large pig carcasses, reported a relatively similar contribution of heath goannas to scavenging, compared with other taxonomic groups (O'Brien et al., 2007). As such, we suggest that regardless of carcass size, heath goannas are important scavengers in coastal mallee ecosystems.

The relative contribution of different taxa to scavenging ecosystem functions is highly seasonally variable in Australia (Bragato et al., 2022; Read & Wilson, 2004; Vandersteen et al., 2023). We carried out this study in early and late summer, when heath goannas undertake most active foraging (Rismiller et al., 2010). This allowed us to assess the maximum potential contribution of heath goannas to scavenging ecosystem functions. We would expect scavenging rates to change throughout the year, with a drop in relative contribution of heath goannas in winter, as their activity levels drop but other species increase reliance on carrion food sources (DeVault et al., 2011; Sawyer et al., 2022; Vandersteen et al., 2023). Future studies should investigate the seasonal variability in scavenging activity in coastal mallee ecosystems.

### 4.2 Effects of vertebrate scavengers on carcass removal and necrophagous fly breeding

Consistent with other studies of semi‐arid ecosystems, we found that carcasses scavenged on by vertebrates lost more mass than those not scavenged on by vertebrates (Parmenter & MacMahon, 2009). This removal of biomass by vertebrate scavengers represents an important ecosystem service to local agricultural communities by preventing the attraction of unwanted pests, limiting the spread of diseases and the damage that unwanted species may cause to livestock, crops, property, and native ecosystems (O'Bryan et al., 2018; Ogada et al., 2012; Peisley et al., 2017).

We demonstrated that carcass scavenging events limited the breeding of necrophagous flies, with a significantly reduced number of maggots in carcasses scavenged on by vertebrates compared with those that were not. The reduction of maggot numbers within carcasses scavenged on by vertebrates is likely a result of the removal of flesh from carcasses by vertebrate scavengers, both reducing the attractiveness of carcasses to egg laying flies and removing food resources for maggots already present in carcasses, leading to an overall reduction of breeding success (Archer & Elgar, 2003; Ullyett, 1950). Furthermore, we observed that carcasses scavenged on, but not completely removed by scavengers (16 of the 61 vertebrate scavenged carcasses) often dried out rapidly as a result of the tearing of carcass skin, and exposure of a greater surface area to desiccation. Dried carcasses appeared to be poor breeding sites for necrophagous flies, possibly because of reduced palatability and fewer gravid female flies discovering carcasses due to a lower volume of attractant chemicals produced (Archer & Elgar, 2003). However, we note that this drying effect may be less marked or important were the study carried out in other seasons.

Our study represents the first step in demonstrating that vertebrate scavenger activity may reduce incidence of flystrike in sheep. Although we did not directly identify flystrike guild fly species (such as *Lucilia cuprina*), we did show that vertebrate scavenging significantly reduces populations of necrophagous flies breeding in carcasses. Studies of Australasian necrophagous flies on sheep pastures have shown that up to 71.6% of flies emerging from carcasses may be members of the flystrike guild (Heath & Appleton, 1999). The removal of carcasses from the vicinity of sheep has therefore been suggested as a potential approach for the control of flystrike (Heath & Appleton, 1999; Smith & Wall, 1997; Wall et al., 1995). Conversely, carcasses may act as “sinks” for the flystrike guild providing alternative breeding sites from live sheep in which high levels of competition with other necrophagous flies may limit breeding success (Heath & Appleton, 1999; Smith & Wall, 1997). The complex interplay of these dynamics requires more study to confirm whether the removal of carrion biomass and associated reduction in necrophagous fly larvae actively reduces the population of flystrike guild species. We suggest a direct study of cases of flystrike in the presence and absence of different densities of carcasses. Flystrike guild flies have high dispersal capacity, with *L. cuprina* dispersing up to 3.5 km in 48 h and a maximum of 17 km after 12 days (Gilmour et al., 1946). As such, carrion removal from outside the boundaries of sheep farms may still have a significant effect in reducing the populations of flystrike guild flies within farms. Hence, the maintenance of large populations of scavenging vertebrates in protected areas and areas of native vegetation as well as on farmlands may have positive spillover effects for farmers.

Necrophagous flies typically develop more slowly in cooler conditions (Bansode et al., 2017) and invertebrate scavenger activity is lower in cooler autumn and winter seasons (DeVault et al., 2004; DeVault et al., 2011; Fust, 2022). Alternatively, vertebrate scavenging activity typically increases during cooler seasons, potentially due to reduced competition with invertebrate scavengers and reduced availability of alternative food sources (DeVault et al., 2011; Sawyer et al., 2022; Vandersteen et al., 2023). As such, the contribution of vertebrates to scavenging ecosystem services is likely to vary seasonally, with an increased relative contribution of vertebrates compared with invertebrates to biomass removal during cooler seasons. Similarly, increased vertebrate scavenging activity during cooler seasons may increase suppression of necrophagous fly populations; however, this may be offset by seasonal reduction of fly breeding rates in response to lower temperatures.

Previous studies have also found that carcass size affects the relative contribution of vertebrate and invertebrate scavengers to carcass removal, whereby smaller carcasses tend to be utilized more completely by vertebrate scavengers than larger carcasses (Fust, 2022; Moleón et al., 2015). Fust (2022) found that vertebrate scavengers had minimal effect on the decay rate of kangaroo carcasses relative to invertebrate scavengers in isolation and suggested that the vertebrate community may have a carcass size threshold at which they are unable to remove additional biomass relative to invertebrates. This threshold may be determined by the size and abundance of vertebrate scavenger species (Fust, 2022). As such, although we found that vertebrate scavenger communities in Australian coastal mallee removed significantly more carcass biomass and significantly reduced numbers of necrophagous flies in rat carcasses, in larger carcasses a size threshold may be reached where vertebrate scavengers cease to have a significant effect on carcass decay. As such, further work is required to assess how vertebrate scavenging contribution varies with carcass size in Australian coastal mallee ecosystems. A lack of large scavenger species (such as dingos and Tasmanian devils) and a low abundance of native mesoscavengers (such as heath goannas) in Australian coastal mallee ecosystems may limit the carcass size threshold at which vertebrate scavengers effectively support scavenging ecosystem services. As such, reintroductions or population augmentations may be necessary to raise this threshold.

### 4.3 Effects of goannas on carcass removal

Areas with more goannas and fewer invasive mammals (KI) had significantly higher levels of carcass removal than areas with fewer goannas and more invasive mammals (SYP), but scavenger community make‐up had no significant effect on number of maggots in a carcass. This suggests that areas with more native scavenger species may contain more individuals providing scavenging ecosystem services and species that are more effective scavengers when compared with invasive mammals.

We found high variance in carcass mass change and maggot density per carcass in intermediate scavenger communities, that is, those with a small heath goanna population alongside cat and fox populations that have been subject to control measures. This high variance may be a result of instability in the scavenger community, linked to invasive mammal control measures. The control of invasive predators can have unintended effects on ecosystems, such as stimulating the demographic release of mesopredator and herbivore populations, disrupting predator social systems causing undesirable behaviors, and stimulating compensatory immigration of different invasive predators (Doherty & Ritchie, 2016). All these factors can paradoxically lead to an increase in negative conservation outcomes stimulated by invasive predator control. In this case, a reduction in the population of foxes and cats could have caused a reduction in scavenging capacity that has not yet been compensated for with a corresponding growth in the goanna population, which may be slow to recover from predation by competition with invasive mammals. Indeed, long‐term monitoring data suggest that the heath goanna population on the SYP has grown in the region between 2014 and 2021 (Jameson et al., in press), but that this has lagged behind the management of the fox and cat population in the south of the region, which started in 2006.

Our data also indicated that carcasses exposed to vertebrates had significantly more mass removed and significantly lower maggot densities per carcass than those exposed to invertebrates alone, but that carcass mass removal and maggot densities per carcass did not differ significantly between carcasses exposed to different vertebrate scavengers. As such, regardless of which vertebrate scavengers can access carcasses, individual vertebrate species are carrying out relatively similar levels of carcass removal and associated necrophagous fly suppression. This suggests that the identity of the scavenging species may not be important in carcass removal and necrophagous fly breeding suppression, indicating a level of redundancy in scavenging ecosystem services carried out by different vertebrate species. This said, all scavenging vertebrates recorded in this study could be broadly classed as mesoscavengers with no records of large‐bodied terrestrial scavengers (such as dingo and Tasmanian devils) or large avian scavengers (such as wedge‐tailed eagles). Previous studies have found limited functional redundancy between vertebrate scavenger guilds (Huijbers et al., 2015). As such, this redundancy may be a reflection of the low functional diversity of scavengers within the study system and results may not be transferable to ecosystems with higher functional diversity.

### 4.4 Management implications

The results of this study suggest that vertebrate scavengers play a significant role in removing carcasses and reducing/limiting the growth of blowfly populations in Australian coastal mallee landscapes. These are important ecosystem services, preventing the attraction of unwanted animals to an area and reducing the spread of disease in populations of native species and livestock. As such, supporting these ecosystem services on the SYP could fulfill rewilding goals within the Marna Banggara Project (Menz & Sharp, 2018). Future planning within the Marna Banggara Project should therefore consider what management actions can be taken to support vertebrate scavenging services.

One important consideration is that invasive predators (cats and foxes) are currently major contributors to scavenging ecosystem services in Australian coastal mallee landscapes. Invasive predators are also major agricultural pests and the main limiting factor in native species recovery (Johnston & Menz, 2019). As such, invasive predators are actively being removed from areas managed for conservation. As they are removed, their contribution to scavenging ecosystem services will be reduced and ultimately lost, as may be already occurring in the intermediate scavenger community treatment studied here. Action should therefore be taken to support native scavenging species that could act as ecological replacements for invasive predators, without having a negative impact on other native species and livestock. The results of this study suggest that the specific identity of vertebrate mesoscavengers may not be important for controlling necrophagous fly numbers. As such, any native vertebrate mesoscavenger may be a suitable replacement for invasive scavengers. The most significant native vertebrate scavengers we identified in this system are corvids (*Corvus mellori*) and heath goannas. As a terrestrial scavenger of similar size to foxes and cats, heath goannas may be the most appropriate ecological replacement.

Current heath goanna populations on the SYP are limited to the southern end of the Marna Banggara Project area, centered around the northern boundary of Dhilba Guuranda‐Innes National Park and Warrenben Conservation Park (Jameson et al., in press). As the heath goanna population on the SYP is very small and likely to be locally endangered (Jameson et al., in press), conservation action to support its growth could benefit landscape‐wide ecosystem services, as well as supporting species‐specific conservation. The current endangered status of the heath goanna on the SYP is likely to be a result of predation and competition by foxes and cats, as such goanna populations may benefit from the removal of foxes and cats from the landscape (Johnston & Menz, 2019). This could lead to natural growth of the local heath goanna population. Supplementary augmentation of heath goanna populations may also be required to grow the population rapidly so that scavenging ecosystem services are not temporarily lost if population recovery is slow.

## 5 CONCLUSIONS

Predation by invasive foxes and cats has been a major cause of native wildlife loss and ecological degradation in Australia. As such, foxes and cats may both contribute to scavenging ecosystem functions themselves and simultaneously reduce the abundance of native scavengers and the functions that they support. This has raised concerns about the relative ability of feral and native scavengers to support scavenging ecosystem services such as the removal of carcasses and suppression of agriculturally harmful blowfly populations. The role that birds and native mammals play as scavengers is well documented, but the importance of squamate scavengers is less well known. This study has shown that the heath goanna is an important native scavenger, along with native birds. In the absence of foxes and cats and where heath goannas were more abundant, carcass removal increased, and necrophagous fly reproduction was suppressed. Removal of foxes and cats from the landscape for ecological restoration is key to rewilding programs in Australia and is likely to increase scavenging ecosystem services by native animals, particularly native squamates. This is the first study we are aware of that explicitly demonstrates the importance of squamates as scavengers in the context of a rewilding approach to ecological restoration.

## AUTHOR CONTRIBUTIONS

**Tom J. M. Jameson:** Conceptualization (lead); data curation (lead); formal analysis (lead); funding acquisition (equal); investigation (lead); methodology (lead); project administration (lead); resources (equal); software (lead); validation (lead); visualization (lead); writing – original draft (lead); writing – review and editing (lead). **Gregory R. Johnston:** Conceptualization (supporting); supervision (supporting); writing – review and editing (supporting). **Max Barr:** Resources (equal); supervision (supporting); writing – review and editing (supporting). **Derek Sandow:** Resources (equal); supervision (supporting); writing – review and editing (supporting). **Jason J. Head:** Conceptualization (supporting); funding acquisition (equal); supervision (supporting); writing – review and editing (supporting). **Edgar C. Turner:** Conceptualization (supporting); supervision (lead); writing – review and editing (supporting).

## CONFLICT OF INTEREST STATEMENT

The authors declare no competing interests.

## Supporting information


Data S1.

## Data Availability

The datasets used and/or analyzed in the present study are available in Table S1 and S2.
