# Supplementary material for: Squamate scavenging services: Heath goannas (Varanus rosenbergi) support carcass removal and may suppress agriculturally damaging blowflies
Source: Ecol Evol. 2024 Jun 25;14(6):e11535. doi: 10.1002/ece3.11535 (PMC11197000; doi:10.1002/ece3.11535)
Supplement: Supplementary file 1 — Data S1. [file ECE3-14-e11535-s001.docx]

**Appendices**

**Table S1.** Raw data for scavenging species count and time taken to find carcass. Treatment refers to the landscape treatment present in the landscape in which the site was located. Scavenging refers to an identified scavenging vertebrate at a site where active scavenging took place (Y = Yes, N = No)

| SiteID | Latitude | Longitude | Taxon | Scavenging | Start date and time | Attack date and time | Time until attack/visit (hours) |
| --- | --- | --- | --- | --- | --- | --- | --- |
| DCP1 | -35.8291 | 137.8851 | *Varanus rosenbergi* | Y | 07/11/2022 10:28 | 07/11/2022 16:39 | 6.18 |
| DCP1 | -35.8291 | 137.8851 | *Varanus rosenbergi* | N | 07/11/2022 10:28 | 08/11/2022 12:35 | 26.12 |
| DCP1 | -35.8291 | 137.8851 | *Varanus rosenbergi* | N | 07/11/2022 10:28 | 08/11/2022 13:21 | 26.88 |
| DCP1 | -35.8291 | 137.8851 | *Varanus rosenbergi* | Y | 07/11/2022 10:28 | 08/11/2022 12:35 | 26.12 |
| DCP1 | -35.8291 | 137.8851 | *Strepera versicolor* | N | 07/11/2022 10:28 | 08/11/2022 10:17 | 23.82 |
| DCP2 | -35.8386 | 137.8846 | *Felis catus* | N | 07/11/2022 11:28 | 11/11/2022 20:24 | 104.93 |
| DCP2 | -35.8386 | 137.8846 | *Varanus rosenbergi* | Y | 07/11/2022 11:28 | 10/11/2022 14:18 | 74.83 |
| DCP3 | -35.8353 | 137.8744 | *Felis catus* | N | 07/11/2022 12:37 | 09/11/2022 04:49 | 40.20 |
| DCP3 | -35.8353 | 137.8744 | *Varanus rosenbergi* | N | 07/11/2022 12:37 | 10/11/2022 12:57 | 72.33 |
| DCP3 | -35.8353 | 137.8744 | *Strepera versicolor* | N | 07/11/2022 12:37 | 07/11/2022 17:23 | 4.77 |
| DCP3 | -35.8353 | 137.8744 | *Strepera versicolor* | N | 07/11/2022 12:37 | 08/11/2022 06:58 | 18.35 |
| DCP3 | -35.8353 | 137.8744 | *Strepera versicolor* | N | 07/11/2022 12:37 | 11/11/2022 12:19 | 95.70 |
| DCP3 | -35.8353 | 137.8744 | *Strepera versicolor* | Y | 07/11/2022 12:37 | 07/11/2022 17:26 | 4.82 |
| DGINP1 | -35.1683 | 136.9166 | *Felis catus* | N | 08/04/2022 10:00 | 10/04/2022 10:42 | 48.70 |
| DGINP1 | -35.1683 | 136.9166 | *Felis catus* | N | 08/04/2022 10:00 | 12/04/2022 01:11 | 87.18 |
| DGINP1 | -35.1683 | 136.9166 | *Felis catus* | Y | 08/04/2022 10:00 | 10/04/2022 10:42 | 48.70 |
| DGINP1 | -35.1683 | 136.9166 | *Strepera versicolor* | N | 08/04/2022 10:00 | 11/04/2022 07:03 | 69.05 |
| DGINP1 | -35.1683 | 136.9166 | *Vulpes vulpes* | N | 19/11/2022 14:00 | 23/11/2022 22:19 | 104.32 |
| DGINP1 | -35.1683 | 136.9166 | *Strepera versicolor* | N | 19/11/2022 14:00 | 20/11/2022 10:52 | 20.87 |
| DGINP1 | -35.1683 | 136.9166 | *Corvus mellori* | N | 08/04/2022 10:00 | 11/04/2022 07:59 | 69.98 |
| DGINP1 | -35.1683 | 136.9166 | *Corvus mellori* | N | 08/04/2022 10:00 | 11/04/2022 08:50 | 70.83 |
| DGINP1 | -35.1683 | 136.9166 | *Corvus mellori* | N | 08/04/2022 10:00 | 12/04/2022 11:21 | 97.35 |
| DGINP1 | -35.1683 | 136.9166 | *Corvus mellori* | Y | 08/04/2022 10:00 | 11/04/2022 08:51 | 70.85 |
| DGINP1 | -35.1683 | 136.9166 | *Corvus mellori* | Y | 19/11/2022 14:00 | 22/11/2022 08:00 | 66.00 |
| DGINP1 | -35.1683 | 136.9166 | *Corvus mellori* | N | 19/11/2022 14:00 | 21/11/2022 15:54 | 49.90 |
| DGINP1 | -35.1683 | 136.9166 | *Corvus mellori* | N | 19/11/2022 14:00 | 21/11/2022 18:14 | 52.23 |
| DGINP1 | -35.1683 | 136.9166 | *Corvus mellori* | N | 19/11/2022 14:00 | 22/11/2022 07:51 | 65.85 |
| DGINP1 | -35.1683 | 136.9166 | *Corvus mellori* | N | 19/11/2022 14:00 | 21/11/2022 18:16 | 52.27 |
| DGINP1 | -35.1683 | 136.9166 | *Tiligua rugosa* | N | 19/11/2022 14:00 | 23/11/2022 12:53 | 94.88 |
| DGINP2 | -35.1872 | 136.9602 | *Felis catus* | N | 19/11/2022 15:00 | 22/11/2022 06:40 | 63.67 |
| DGINP2 | -35.1872 | 136.9602 | *Felis catus* | N | 19/11/2022 15:00 | 23/11/2022 19:15 | 100.25 |
| DGINP2 | -35.1872 | 136.9602 | *Felis catus* | N | 19/11/2022 15:00 | 23/11/2022 22:43 | 103.72 |
| DGINP2 | -35.1872 | 136.9602 | *Felis catus* | Y | 19/11/2022 15:00 | 23/11/2022 19:25 | 100.42 |
| DGINP2 | -35.1872 | 136.9602 | *Felis catus* | N | 19/11/2022 15:00 | 23/11/2022 22:47 | 103.78 |
| DGINP2 | -35.1872 | 136.9602 | *Corvus mellori* | N | 08/04/2022 11:00 | 11/04/2022 15:15 | 76.25 |
| DGINP2 | -35.1872 | 136.9602 | *Tiligua rugosa* | N | 19/11/2022 15:00 | 23/11/2022 12:29 | 93.48 |
| DGINP2 | -35.1872 | 136.9602 | *Podargus strigoides* | Y | 08/04/2022 11:00 | 12/04/2022 01:53 | 86.88 |
| DGINP2 | -35.1872 | 136.9602 | *Podargus strigoides* | Y | 08/04/2022 11:00 | 12/04/2022 02:54 | 87.90 |
| DGINP3 | -35.2313 | 136.946 | *Strepera versicolor* | N | 08/04/2022 12:45 | 09/04/2022 13:39 | 24.90 |
| DGINP3 | -35.2313 | 136.946 | *Corvus mellori* | N | 08/04/2022 12:45 | 08/04/2022 16:53 | 4.13 |
| DGINP3 | -35.2313 | 136.946 | *Corvus mellori* | N | 08/04/2022 12:45 | 08/04/2022 17:28 | 4.72 |
| DGINP3 | -35.2313 | 136.946 | *Corvus mellori* | N | 08/04/2022 12:45 | 10/04/2022 09:07 | 44.37 |
| DGINP3 | -35.2313 | 136.946 | *Corvus mellori* | N | 08/04/2022 12:45 | 10/04/2022 12:50 | 48.08 |
| DGINP3 | -35.2313 | 136.946 | *Corvus mellori* | N | 08/04/2022 12:45 | 10/04/2022 13:29 | 48.73 |
| DGINP3 | -35.2313 | 136.946 | *Corvus mellori* | N | 08/04/2022 12:45 | 11/04/2022 08:58 | 68.22 |
| DGINP3 | -35.2313 | 136.946 | *Corvus mellori* | N | 08/04/2022 12:45 | 11/04/2022 09:29 | 68.73 |
| DGINP3 | -35.2313 | 136.946 | *Corvus mellori* | Y | 08/04/2022 12:45 | 08/04/2022 16:53 | 4.13 |
| DGINP3.1 | -35.2313 | 136.946 | *Felis catus* | N | 19/11/2022 16:00 | 23/11/2022 05:32 | 85.53 |
| DGINP3.1 | -35.2313 | 136.946 | *Strepera versicolor* | N | 19/11/2022 16:00 | 21/11/2022 17:08 | 49.13 |
| DGINP3.1 | -35.2313 | 136.946 | *Strepera versicolor* | N | 19/11/2022 16:00 | 22/11/2022 15:07 | 71.12 |
| DGINP3.1 | -35.2313 | 136.946 | *Corvus mellori* | Y | 19/11/2022 16:00 | 21/11/2022 19:17 | 51.28 |
| DGINP3.1 | -35.2313 | 136.946 | *Corvus mellori* | Y | 19/11/2022 16:00 | 22/11/2022 07:05 | 63.08 |
| DGINP3.1 | -35.2313 | 136.946 | *Corvus mellori* | Y | 19/11/2022 16:00 | 22/11/2022 09:50 | 65.83 |
| DGINP3.1 | -35.2313 | 136.946 | *Corvus mellori* | Y | 19/11/2022 16:00 | 22/11/2022 14:05 | 70.08 |
| DGINP3.1 | -35.2313 | 136.946 | *Corvus mellori* | N | 19/11/2022 16:00 | 24/11/2022 11:09 | 115.15 |
| DGINP3.1 | -35.2313 | 136.946 | *Corvus mellori* | N | 19/11/2022 16:00 | 21/11/2022 17:34 | 49.57 |
| DGINP3.1 | -35.2313 | 136.946 | *Corvus mellori* | N | 19/11/2022 16:00 | 21/11/2022 18:45 | 50.75 |
| DGINP3.1 | -35.2313 | 136.946 | *Corvus mellori* | N | 19/11/2022 16:00 | 22/11/2022 09:37 | 65.62 |
| DGINP3.1 | -35.2313 | 136.946 | *Corvus mellori* | N | 19/11/2022 16:00 | 22/11/2022 10:32 | 66.53 |
| DGINP3.1 | -35.2313 | 136.946 | *Corvus mellori* | Y | 19/11/2022 16:00 | 22/11/2022 12:18 | 68.30 |
| DGINP3.1 | -35.2313 | 136.946 | *Corvus mellori* | N | 19/11/2022 16:00 | 22/11/2022 14:01 | 70.02 |
| DGINP3.1 | -35.2313 | 136.946 | *Corvus mellori* | Y | 19/11/2022 16:00 | 22/11/2022 14:25 | 70.42 |
| DGINP3.1 | -35.2313 | 136.946 | *Corvus mellori* | Y | 19/11/2022 16:00 | 22/11/2022 15:08 | 71.13 |
| DGINP3.1 | -35.2313 | 136.946 | *Corvus mellori* | Y | 19/11/2022 16:00 | 22/11/2022 17:32 | 73.53 |
| DGINP3.1 | -35.2313 | 136.946 | *Corvus mellori* | Y | 19/11/2022 16:00 | 23/11/2022 17:04 | 97.07 |
| DGINP3.1 | -35.2313 | 136.946 | *Corvus mellori* | Y | 19/11/2022 16:00 | 21/11/2022 16:57 | 48.95 |
| DGINP3.1 | -35.2313 | 136.946 | *Corvus mellori* | Y | 19/11/2022 16:00 | 21/11/2022 18:01 | 50.02 |
| DGINP3.1 | -35.2313 | 136.946 | *Corvus mellori* | Y | 19/11/2022 16:00 | 21/11/2022 18:44 | 50.73 |
| DGINP3.1 | -35.2313 | 136.946 | *Corvus mellori* | N | 19/11/2022 16:00 | 22/11/2022 07:17 | 63.28 |
| DGINP3.1 | -35.2313 | 136.946 | *Corvus mellori* | N | 19/11/2022 16:00 | 22/11/2022 09:36 | 65.60 |
| DGINP3.1 | -35.2313 | 136.946 | *Corvus mellori* | N | 19/11/2022 16:00 | 22/11/2022 10:32 | 66.53 |
| DGINP3.1 | -35.2313 | 136.946 | *Corvus mellori* | N | 19/11/2022 16:00 | 22/11/2022 15:07 | 71.12 |
| DGINP3.1 | -35.2313 | 136.946 | *Corvus mellori* | N | 19/11/2022 16:00 | 22/11/2022 16:04 | 72.07 |
| DGINP3.1 | -35.2313 | 136.946 | *Tiligua rugosa* | Y | 19/11/2022 16:00 | 22/11/2022 14:01 | 70.02 |
| EP1 | -35.7745 | 138.055 | *Corvus mellori* | Y | 06/11/2022 14:51 | 07/11/2022 07:10 | 16.32 |
| EP1 | -35.7745 | 138.055 | *Corvus mellori* | N | 06/11/2022 14:51 | 08/11/2022 09:08 | 42.28 |
| EP1 | -35.7745 | 138.055 | *Corvus mellori* | N | 06/11/2022 14:51 | 09/11/2022 06:15 | 63.40 |
| EP1 | -35.7745 | 138.055 | *Corvus mellori* | Y | 06/11/2022 14:51 | 07/11/2022 07:06 | 16.25 |
| EP1 | -35.7745 | 138.055 | *Corvus mellori* | N | 06/11/2022 14:51 | 07/11/2022 10:00 | 19.15 |
| EP1 | -35.7745 | 138.055 | *Corvus mellori* | Y | 06/11/2022 14:51 | 07/11/2022 08:27 | 17.60 |
| EP1 | -35.7745 | 138.055 | *Corvus mellori* | Y | 06/11/2022 14:51 | 07/11/2022 09:44 | 18.88 |
| EP1 | -35.7745 | 138.055 | *Corvus mellori* | Y | 06/11/2022 14:51 | 07/11/2022 10:57 | 20.10 |
| EP1 | -35.7745 | 138.055 | *Corvus mellori* | Y | 06/11/2022 14:51 | 08/11/2022 06:08 | 39.28 |
| EP1 | -35.7745 | 138.055 | *Corvus mellori* | Y | 06/11/2022 14:51 | 08/11/2022 08:08 | 41.28 |
| EP1 | -35.7745 | 138.055 | *Corvus mellori* | Y | 06/11/2022 14:51 | 09/11/2022 06:04 | 63.22 |
| EP1 | -35.7745 | 138.055 | *Corvus mellori* | Y | 06/11/2022 14:51 | 09/11/2022 06:59 | 64.13 |
| EP1 | -35.7745 | 138.055 | *Corvus mellori* | Y | 06/11/2022 14:51 | 10/11/2022 07:39 | 88.80 |
| EP1 | -35.7745 | 138.055 | *Corvus mellori* | Y | 06/11/2022 14:51 | 10/11/2022 17:08 | 98.28 |
| EP2 | -35.7839 | 138.0639 | *Corvus mellori* | N | 06/11/2022 15:36 | 09/11/2022 12:34 | 68.97 |
| EP2 | -35.7839 | 138.0639 | *Corvus mellori* | N | 06/11/2022 15:36 | 09/11/2022 15:59 | 72.38 |
| EP2 | -35.7839 | 138.0639 | *Corvus mellori* | N | 06/11/2022 15:36 | 10/11/2022 07:53 | 88.28 |
| EP2 | -35.7839 | 138.0639 | *Corvus mellori* | Y | 06/11/2022 15:36 | 09/11/2022 15:59 | 72.38 |
| EP2 | -35.7839 | 138.0639 | *Corvus mellori* | Y | 06/11/2022 15:36 | 09/11/2022 15:59 | 72.38 |
| EP3 | -35.7994 | 138.0674 | *Felis catus* | N | 06/11/2022 16:22 | 06/11/2022 21:41 | 5.32 |
| EP3 | -35.7994 | 138.0674 | *Varanus rosenbergi* | Y | 06/11/2022 16:22 | 07/11/2022 09:27 | 17.08 |
| EP3 | -35.7994 | 138.0674 | *Varanus rosenbergi* | N | 06/11/2022 16:22 | 07/11/2022 09:27 | 17.08 |
| EP3 | -35.7994 | 138.0674 | *Varanus rosenbergi* | Y | 06/11/2022 16:22 | 07/11/2022 17:17 | 24.92 |
| EP3 | -35.7994 | 138.0674 | *Corvus mellori* | Y | 06/11/2022 16:22 | 09/11/2022 09:12 | 64.83 |
| EP3 | -35.7994 | 138.0674 | *Corvus mellori* | N | 06/11/2022 16:22 | 09/11/2022 09:12 | 64.83 |
| EP3 | -35.7994 | 138.0674 | *Corvus mellori* | N | 06/11/2022 16:22 | 10/11/2022 10:50 | 90.47 |
| EP3 | -35.7994 | 138.0674 | *Gymnorhina tibicen* | N | 06/11/2022 16:22 | 07/11/2022 12:55 | 20.55 |
| FB2 | -35.176 | 137.1749 | *Corvus mellori* | N | 27/03/2022 11:00 | 28/03/2022 08:17 | 21.28 |
| FB3 | -35.1811 | 137.185 | *Felis catus* | N | 18/11/2022 10:45 | 21/11/2022 04:32 | 65.78 |
| FB3 | -35.1811 | 137.185 | *Strepera versicolor* | N | 18/11/2022 10:45 | 18/11/2022 15:21 | 4.60 |
| FB3 | -35.1811 | 137.185 | *Strepera versicolor* | N | 18/11/2022 10:45 | 18/11/2022 19:51 | 9.10 |
| FB3 | -35.1811 | 137.185 | *Strepera versicolor* | N | 18/11/2022 10:45 | 18/11/2022 15:21 | 4.60 |
| FB3 | -35.1811 | 137.185 | *Strepera versicolor* | N | 18/11/2022 10:45 | 18/11/2022 19:53 | 9.13 |
| FB3 | -35.1811 | 137.185 | *Gymnorhina tibicen* | N | 18/11/2022 10:45 | 18/11/2022 16:50 | 6.08 |
| FB3 | -35.1811 | 137.185 | *Gymnorhina tibicen* | N | 18/11/2022 10:45 | 18/11/2022 16:51 | 6.10 |
| LB1 | -34.9133 | 137.1844 | *Felis catus* | N | 24/03/2022 10:00 | 26/03/2022 01:43 | 39.72 |
| LB1 | -34.9133 | 137.1844 | *Corvus mellori* | N | 24/03/2022 10:00 | 27/03/2022 08:56 | 70.93 |
| LB1 | -34.9133 | 137.1844 | *Corvus mellori* | Y | 24/03/2022 10:00 | 25/03/2022 07:44 | 21.73 |
| LB1 | -34.9133 | 137.1844 | *Corvus mellori* | Y | 24/03/2022 10:00 | 25/03/2022 10:51 | 24.85 |
| LB1 | -34.9133 | 137.1844 | *Corvus mellori* | Y | 20/10/2022 10:00 | 20/10/2022 12:31 | 2.52 |
| LB1 | -34.9133 | 137.1844 | *Corvus mellori* | Y | 20/10/2022 10:00 | 20/10/2022 14:57 | 4.95 |
| LB1 | -34.9133 | 137.1844 | *Corvus mellori* | Y | 20/10/2022 10:00 | 20/10/2022 15:13 | 5.22 |
| LB1 | -34.9133 | 137.1844 | *Corvus mellori* | Y | 20/10/2022 10:00 | 20/10/2022 12:05 | 2.08 |
| LB1 | -34.9133 | 137.1844 | *Corvus mellori* | Y | 20/10/2022 10:00 | 20/10/2022 13:38 | 3.63 |
| LB1 | -34.9133 | 137.1844 | *Corvus mellori* | N | 20/10/2022 10:00 | 20/10/2022 14:32 | 4.53 |
| LB1 | -34.9133 | 137.1844 | *Corvus mellori* | N | 20/10/2022 10:00 | 21/10/2022 14:36 | 28.60 |
| LB1 | -34.9133 | 137.1844 | *Corvus mellori* | N | 20/10/2022 10:00 | 23/10/2022 22:20 | 84.33 |
| LB1 | -34.9133 | 137.1844 | *Corvus mellori* | N | 20/10/2022 10:00 | 20/10/2022 12:09 | 2.15 |
| LB1 | -34.9133 | 137.1844 | *Corvus mellori* | N | 20/10/2022 10:00 | 20/10/2022 17:09 | 7.15 |
| LB1 | -34.9133 | 137.1844 | *Corvus mellori* | N | 20/10/2022 10:00 | 21/10/2022 09:04 | 23.07 |
| LB1 | -34.9133 | 137.1844 | *Corvus mellori* | N | 20/10/2022 10:00 | 21/10/2022 17:28 | 31.47 |
| LB1 | -34.9133 | 137.1844 | *Corvus mellori* | N | 20/10/2022 10:00 | 22/10/2022 16:33 | 54.55 |
| LB1 | -34.9133 | 137.1844 | *Corvus mellori* | N | 20/10/2022 10:00 | 22/10/2022 17:29 | 55.48 |
| LB1 | -34.9133 | 137.1844 | *Corvus mellori* | N | 20/10/2022 10:00 | 23/10/2022 10:20 | 72.33 |
| LB2 | -34.9134 | 137.2059 | *Corvus coronoides* | Y | 20/10/2022 10:43 | 21/10/2022 08:27 | 21.73 |
| LB2 | -34.9134 | 137.2059 | *Corvus coronoides* | Y | 20/10/2022 10:43 | 21/10/2022 17:32 | 30.82 |
| LB2 | -34.9134 | 137.2059 | *Corvus coronoides* | Y | 20/10/2022 10:43 | 22/10/2022 07:16 | 44.55 |
| LB2 | -34.9134 | 137.2059 | *Corvus coronoides* | Y | 20/10/2022 10:43 | 22/10/2022 09:05 | 46.37 |
| LB2 | -34.9134 | 137.2059 | *Corvus coronoides* | Y | 20/10/2022 10:43 | 24/10/2022 06:46 | 92.05 |
| LB2 | -34.9134 | 137.2059 | *Felis catus* | N | 24/03/2022 10:30 | 25/03/2022 03:10 | 16.67 |
| LB2 | -34.9134 | 137.2059 | *Vulpes vulpes* | Y | 24/03/2022 10:30 | 25/03/2022 19:09 | 32.65 |
| LB2 | -34.9134 | 137.2059 | *Corvus mellori* | N | 24/03/2022 10:30 | 25/03/2022 11:11 | 24.68 |
| LB2 | -34.9134 | 137.2059 | *Corvus mellori* | N | 24/03/2022 10:30 | 25/03/2022 12:43 | 26.22 |
| LB2 | -34.9134 | 137.2059 | *Corvus mellori* | N | 24/03/2022 10:30 | 25/03/2022 13:12 | 26.70 |
| LB2 | -34.9134 | 137.2059 | *Corvus mellori* | Y | 24/03/2022 10:30 | 25/03/2022 11:04 | 24.57 |
| LB2 | -34.9134 | 137.2059 | *Corvus mellori* | Y | 24/03/2022 10:30 | 25/03/2022 12:00 | 25.50 |
| LB2 | -34.9134 | 137.2059 | *Corvus mellori* | Y | 24/03/2022 10:30 | 25/03/2022 12:44 | 26.23 |
| LB2 | -34.9134 | 137.2059 | *Corvus mellori* | Y | 24/03/2022 10:30 | 26/03/2022 08:10 | 45.67 |
| LB2 | -34.9134 | 137.2059 | *Corvus mellori* | Y | 24/03/2022 10:30 | 26/03/2022 09:34 | 47.07 |
| LB2 | -34.9134 | 137.2059 | *Corvus mellori* | Y | 24/03/2022 10:30 | 26/03/2022 09:40 | 47.17 |
| LB2 | -34.9134 | 137.2059 | *Corvus mellori* | Y | 20/10/2022 10:43 | 20/10/2022 19:19 | 8.60 |
| LB2 | -34.9134 | 137.2059 | *Corvus mellori* | Y | 20/10/2022 10:43 | 21/10/2022 06:45 | 20.03 |
| LB2 | -34.9134 | 137.2059 | *Corvus mellori* | Y | 20/10/2022 10:43 | 21/10/2022 13:27 | 26.73 |
| LB2 | -34.9134 | 137.2059 | *Corvus mellori* | Y | 20/10/2022 10:43 | 20/10/2022 19:19 | 8.60 |
| LB2 | -34.9134 | 137.2059 | *Corvus mellori* | N | 20/10/2022 10:43 | 22/10/2022 08:08 | 45.42 |
| LB2 | -34.9134 | 137.2059 | *Corvus mellori* | N | 20/10/2022 10:43 | 22/10/2022 13:37 | 50.90 |
| LB2 | -34.9134 | 137.2059 | *Corvus mellori* | N | 20/10/2022 10:43 | 24/10/2022 06:46 | 92.05 |
| LB3 | -34.9072 | 137.2264 | *Corvus mellori* | N | 20/10/2022 11:45 | 20/10/2022 13:05 | 1.33 |
| LB3 | -34.9072 | 137.2264 | *Corvus mellori* | N | 20/10/2022 11:45 | 21/10/2022 08:39 | 20.90 |
| LB3 | -34.9072 | 137.2264 | *Corvus mellori* | N | 20/10/2022 11:45 | 21/10/2022 16:29 | 28.73 |
| LB3 | -34.9072 | 137.2264 | *Corvus mellori* | Y | 20/10/2022 11:45 | 22/10/2022 07:41 | 43.93 |
| LB3 | -34.9072 | 137.2264 | *Corvus mellori* | Y | 20/10/2022 11:45 | 22/10/2022 09:07 | 45.37 |
| LB3 | -34.9072 | 137.2264 | *Corvus mellori* | Y | 20/10/2022 11:45 | 22/10/2022 10:14 | 46.48 |
| LB3 | -34.9072 | 137.2264 | *Corvus mellori* | Y | 20/10/2022 11:45 | 22/10/2022 11:10 | 47.42 |
| LB3 | -34.9072 | 137.2264 | *Corvus mellori* | Y | 20/10/2022 11:45 | 22/10/2022 14:17 | 50.53 |
| LB3 | -34.9072 | 137.2264 | *Corvus mellori* | Y | 20/10/2022 11:45 | 22/10/2022 16:32 | 52.78 |
| LB3 | -34.9072 | 137.2264 | *Corvus mellori* | Y | 20/10/2022 11:45 | 23/10/2022 13:40 | 73.92 |
| LB3 | -34.9072 | 137.2264 | *Corvus mellori* | Y | 20/10/2022 11:45 | 24/10/2022 09:27 | 93.70 |
| LB3 | -34.9072 | 137.2264 | *Corvus mellori* | Y | 20/10/2022 11:45 | 25/10/2022 10:23 | 118.63 |
| LB3 | -34.9072 | 137.2264 | *Corvus mellori* | N | 20/10/2022 11:45 | 20/10/2022 13:10 | 1.42 |
| LB3 | -34.9072 | 137.2264 | *Corvus mellori* | N | 20/10/2022 11:45 | 23/10/2022 07:29 | 67.73 |
| LB3 | -34.9072 | 137.2264 | *Corvus coronoides* | N | 20/10/2022 11:45 | 24/10/2022 09:29 | 93.73 |
| LB3 | -34.9072 | 137.2264 | *Vulpes vulpes* | Y | 24/03/2022 11:30 | 29/03/2022 07:09 | 115.65 |
| LB3 | -34.9072 | 137.2264 | *Corvus mellori* | N | 24/03/2022 11:30 | 25/03/2022 10:00 | 22.50 |
| LB3 | -34.9072 | 137.2264 | *Corvus mellori* | N | 24/03/2022 11:30 | 27/03/2022 08:37 | 69.12 |
| LB3 | -34.9072 | 137.2264 | *Corvus mellori* | N | 20/10/2022 11:45 | 24/10/2022 09:22 | 93.62 |
| LB3 | -34.9072 | 137.2264 | *Corvus mellori* | N | 20/10/2022 11:45 | 25/10/2022 10:55 | 119.17 |
| LB3 | -34.9072 | 137.2264 | *Corvus mellori* | Y | 20/10/2022 11:45 | 21/10/2022 10:23 | 22.63 |
| LB3 | -34.9072 | 137.2264 | *Tiligua rugosa* | N | 20/10/2022 11:45 | 20/10/2022 17:17 | 5.53 |
| PD1 | -35.1507 | 137.339 | *Vulpes vulpes* | N | 07/04/2022 10:30 | 08/04/2022 06:18 | 19.80 |
| PD1 | -35.1507 | 137.339 | *Vulpes vulpes* | Y | 07/04/2022 10:30 | 08/04/2022 06:18 | 19.80 |
| PD1 | -35.1507 | 137.339 | *Corvus mellori* | Y | 17/11/2022 10:30 | 18/11/2022 14:23 | 27.88 |
| PD1 | -35.1507 | 137.339 | *Corvus mellori* | Y | 17/11/2022 10:30 | 18/11/2022 16:19 | 29.82 |
| PD1 | -35.1507 | 137.339 | *Corvus mellori* | Y | 17/11/2022 10:30 | 18/11/2022 18:00 | 31.50 |
| PD1 | -35.1507 | 137.339 | *Corvus mellori* | Y | 17/11/2022 10:30 | 19/11/2022 09:35 | 47.08 |
| PD1 | -35.1507 | 137.339 | *Corvus mellori* | N | 17/11/2022 10:30 | 20/11/2022 07:23 | 68.88 |
| PD1 | -35.1507 | 137.339 | *Corvus mellori* | N | 17/11/2022 10:30 | 18/11/2022 14:31 | 28.02 |
| PD1 | -35.1507 | 137.339 | *Corvus mellori* | N | 17/11/2022 10:30 | 18/11/2022 16:32 | 30.03 |
| PD1 | -35.1507 | 137.339 | *Corvus mellori* | N | 17/11/2022 10:30 | 19/11/2022 09:35 | 47.08 |
| PD1 | -35.1507 | 137.339 | *Corvus mellori* | N | 17/11/2022 10:30 | 20/11/2022 07:20 | 68.83 |
| PD1 | -35.1507 | 137.339 | *Corvus mellori* | Y | 17/11/2022 10:30 | 18/11/2022 14:03 | 27.55 |
| PD1 | -35.1507 | 137.339 | *Corvus mellori* | Y | 17/11/2022 10:30 | 18/11/2022 16:06 | 29.60 |

**Table S2.** Raw data for carcass mass change and necrophagous fly breeding response variables. Treatment refers to the landscape treatment present in the landscape in which the site was located. Vertebrate scavenging refers to whether or not a vertebrate actively scavenged on a carcass. Vertebrate scavenger taxon refers to the taxon that actively undertook scavenging where scavenging did occur. Maggot number refers to the number of maggots in a carcass after the exposure period.

| SiteID | Latitude | Longitude | Location | Date started | Date ended | Time started | Time ended | Treatment | Exclosure | Vertebrate scavenging | Vertebrate scavenger taxon | Mass start (g) | Mass end (g) | Mass change (g) | Maggot number |
| --- | --- | --- | --- | --- | --- | --- | --- | --- | --- | --- | --- | --- | --- | --- | --- |
| DCP1 | -35.8291 | 137.8851 | Dudley Conservation Park | 07/11/2022 | 12/11/2022 | 10:28 | 09:35 | Native | Accessible to all | Scavenging | Squamate | 123 | 0 | 123 | 0 |
| DCP1 | -35.8291 | 137.8851 | Dudley Conservation Park | 07/11/2022 | 12/11/2022 | 10:28 | 09:35 | Native | Birds only | No Scavenging | N/A | 159 | 59 | 100 | 267 |
| DCP1 | -35.8291 | 137.8851 | Dudley Conservation Park | 07/11/2022 | 12/11/2022 | 10:28 | 09:35 | Native | Invertebrates only | No Scavenging | N/A | 128 | 40 | 88 | 2 |
| DCP1 | -35.8291 | 137.8851 | Dudley Conservation Park | 07/11/2022 | 12/11/2022 | 10:28 | 09:35 | Native | Squamates only | Scavenging | Squamate | 147 | 0 | 147 | 0 |
| DCP2 | -35.8386 | 137.8846 | Dudley Conservation Park | 07/11/2022 | 12/11/2022 | 11:28 | 10:30 | Native | Accessible to all | No Scavenging | N/A | 198 | 102 | 96 | 142 |
| DCP2 | -35.8386 | 137.8846 | Dudley Conservation Park | 07/11/2022 | 12/11/2022 | 11:28 | 10:30 | Native | Birds only | No Scavenging | N/A | 122 | 34 | 88 | 73 |
| DCP2 | -35.8386 | 137.8846 | Dudley Conservation Park | 07/11/2022 | 12/11/2022 | 11:28 | 10:30 | Native | Invertebrates only | No Scavenging | N/A | 192 | 67 | 125 | 11 |
| DCP2 | -35.8386 | 137.8846 | Dudley Conservation Park | 07/11/2022 | 12/11/2022 | 11:28 | 10:30 | Native | Squamates only | Scavenging | Squamate | 189 | 2 | 187 | 0 |
| DCP3 | -35.8353 | 137.8744 | Dudley Conservation Park | 07/11/2022 | 12/11/2022 | 12:37 | 11:30 | Native | Accessible to all | Scavenging | Bird | 163 | 43 | 120 | 2 |
| DCP3 | -35.8353 | 137.8744 | Dudley Conservation Park | 07/11/2022 | 12/11/2022 | 12:37 | 11:30 | Native | Birds only | No Scavenging | N/A | 169 | 57 | 112 | 47 |
| DCP3 | -35.8353 | 137.8744 | Dudley Conservation Park | 07/11/2022 | 12/11/2022 | 12:37 | 11:30 | Native | Invertebrates only | No Scavenging | N/A | 184 | 54 | 130 | 66 |
| DCP3 | -35.8353 | 137.8744 | Dudley Conservation Park | 07/11/2022 | 12/11/2022 | 12:37 | 11:30 | Native | Squamates only | No Scavenging | N/A | 184 | 51 | 133 | 1141 |
| DGINP1 | -35.1683 | 136.9166 | Dhilba Guuranda-Innes National Park | 08/04/2022 | 13/04/2022 | 10:00 | 10:30 | Intermediate | Accessible to all | Scavenging | Mammal | 147 | 0 | 147 | 0 |
| DGINP1 | -35.1683 | 136.9166 | Dhilba Guuranda-Innes National Park | 19/11/2022 | 24/11/2022 | 14:00 | 10:10 | Intermediate | Accessible to all | No Scavenging | N/A | 131 | 111 | 20 | 80 |
| DGINP1 | -35.1683 | 136.9166 | Dhilba Guuranda-Innes National Park | 08/04/2022 | 13/04/2022 | 10:00 | 10:30 | Intermediate | Birds only | Scavenging | Bird | 163 | 0 | 163 | 0 |
| DGINP1 | -35.1683 | 136.9166 | Dhilba Guuranda-Innes National Park | 19/11/2022 | 24/11/2022 | 14:00 | 10:10 | Intermediate | Birds only | Scavenging | Bird | 130 | 0 | 130 | 0 |
| DGINP1 | -35.1683 | 136.9166 | Dhilba Guuranda-Innes National Park | 08/04/2022 | 13/04/2022 | 10:00 | 10:30 | Intermediate | Invertebrates only | No Scavenging | N/A | 127 | 90 | 37 | 290 |
| DGINP1 | -35.1683 | 136.9166 | Dhilba Guuranda-Innes National Park | 19/11/2022 | 24/11/2022 | 14:00 | 10:10 | Intermediate | Invertebrates only | No Scavenging | N/A | 145 | 95 | 50 | 176 |
| DGINP1 | -35.1683 | 136.9166 | Dhilba Guuranda-Innes National Park | 08/04/2022 | 13/04/2022 | 10:00 | 10:30 | Intermediate | Squamates only | No Scavenging | N/A | 155 | 104 | 51 | 325 |
| DGINP1 | -35.1683 | 136.9166 | Dhilba Guuranda-Innes National Park | 19/11/2022 | 24/11/2022 | 14:00 | 10:10 | Intermediate | Squamates only | No Scavenging | N/A | 154 | 101 | 53 | 41 |
| DGINP2 | -35.1872 | 136.9602 | Dhilba Guuranda-Innes National Park | 08/04/2022 | 13/04/2022 | 11:00 | 11:30 | Intermediate | Accessible to all | No Scavenging | N/A | 65 | 10 | 55 | 7 |
| DGINP2 | -35.1872 | 136.9602 | Dhilba Guuranda-Innes National Park | 19/11/2022 | 24/11/2022 | 15:00 | 11:00 | Intermediate | Accessible to all | Scavenging | Mammal | 152 | 0 | 152 | 0 |
| DGINP2 | -35.1872 | 136.9602 | Dhilba Guuranda-Innes National Park | 08/04/2022 | 13/04/2022 | 11:00 | 11:30 | Intermediate | Birds only | Scavenging | Bird | 62 | 10 | 52 | 24 |
| DGINP2 | -35.1872 | 136.9602 | Dhilba Guuranda-Innes National Park | 19/11/2022 | 24/11/2022 | 15:00 | 11:00 | Intermediate | Birds only | No Scavenging | N/A | 157 | 147 | 10 | 64 |
| DGINP2 | -35.1872 | 136.9602 | Dhilba Guuranda-Innes National Park | 08/04/2022 | 13/04/2022 | 11:00 | 11:30 | Intermediate | Invertebrates only | No Scavenging | N/A | 68 | 20 | 48 | 130 |
| DGINP2 | -35.1872 | 136.9602 | Dhilba Guuranda-Innes National Park | 19/11/2022 | 24/11/2022 | 15:00 | 11:00 | Intermediate | Invertebrates only | No Scavenging | N/A | 170 | 144 | 26 | 102 |
| DGINP2 | -35.1872 | 136.9602 | Dhilba Guuranda-Innes National Park | 08/04/2022 | 13/04/2022 | 11:00 | 11:30 | Intermediate | Squamates only | No Scavenging | N/A | 75 | 25 | 50 | 94 |
| DGINP2 | -35.1872 | 136.9602 | Dhilba Guuranda-Innes National Park | 19/11/2022 | 24/11/2022 | 15:00 | 11:00 | Intermediate | Squamates only | No Scavenging | N/A | 148 | 124 | 24 | 9 |
| DGINP3 | -35.2313 | 136.946 | Dhilba Guuranda-Innes National Park | 08/04/2022 | 13/04/2022 | 12:15 | 12:15 | Intermediate | Accessible to all | Scavenging | Bird | 140 | 0 | 140 | 0 |
| DGINP3 | -35.2313 | 136.946 | Dhilba Guuranda-Innes National Park | 08/04/2022 | 13/04/2022 | 12:15 | 12:15 | Intermediate | Birds only | No Scavenging | N/A | 130 | 65 | 65 | 450 |
| DGINP3 | -35.2313 | 136.946 | Dhilba Guuranda-Innes National Park | 08/04/2022 | 13/04/2022 | 12:15 | 12:15 | Intermediate | Invertebrates only | No Scavenging | N/A | 147 | 5 | 142 | 0 |
| DGINP3 | -35.2313 | 136.946 | Dhilba Guuranda-Innes National Park | 08/04/2022 | 13/04/2022 | 12:15 | 12:15 | Intermediate | Squamates only | No Scavenging | N/A | 135 | 25 | 110 | 250 |
| DGINP3.1 | -35.2313 | 136.946 | Dhilba Guuranda-Innes National Park | 19/11/2022 | 24/11/2022 | 16:00 | 12:00 | Intermediate | Accessible to all | Scavenging | Bird | 117 | 0 | 117 | 0 |
| DGINP3.1 | -35.2313 | 136.946 | Dhilba Guuranda-Innes National Park | 19/11/2022 | 24/11/2022 | 16:00 | 12:00 | Intermediate | Birds only | Scavenging | Bird | 135 | 0 | 135 | 0 |
| DGINP3.1 | -35.2313 | 136.946 | Dhilba Guuranda-Innes National Park | 19/11/2022 | 24/11/2022 | 16:00 | 12:00 | Intermediate | Invertebrates only | No Scavenging | N/A | 118 | 76 | 42 | 325 |
| DGINP3.1 | -35.2313 | 136.946 | Dhilba Guuranda-Innes National Park | 19/11/2022 | 24/11/2022 | 16:00 | 12:00 | Intermediate | Squamates only | Scavenging | Squamate | 164 | 20 | 144 | 0 |
| EP1 | -35.7745 | 138.055 | Lashmar Conservation Park | 06/11/2022 | 11/11/2022 | 14:51 | 10:00 | Native | Accessible to all | Scavenging | Bird | 164 | 0 | 164 | 0 |
| EP1 | -35.7745 | 138.055 | Lashmar Conservation Park | 06/11/2022 | 11/11/2022 | 14:51 | 10:00 | Native | Birds only | Scavenging | Bird | 122 | 0 | 122 | 0 |
| EP1 | -35.7745 | 138.055 | Lashmar Conservation Park | 06/11/2022 | 11/11/2022 | 14:51 | 10:00 | Native | Invertebrates only | No Scavenging | N/A | 123 | 20 | 103 | 46 |
| EP1 | -35.7745 | 138.055 | Lashmar Conservation Park | 06/11/2022 | 11/11/2022 | 14:51 | 10:00 | Native | Squamates only | Scavenging | Bird | 129 | 6 | 123 | 0 |
| EP2 | -35.7839 | 138.0639 | Lashmar Conservation Park | 06/11/2022 | 11/11/2022 | 15:36 | 10:30 | Native | Accessible to all | Scavenging | Bird | 149 | 0 | 149 | 0 |
| EP2 | -35.7839 | 138.0639 | Lashmar Conservation Park | 06/11/2022 | 11/11/2022 | 15:36 | 10:30 | Native | Birds only | Scavenging | Bird | 143 | 0 | 143 | 0 |
| EP2 | -35.7839 | 138.0639 | Lashmar Conservation Park | 06/11/2022 | 11/11/2022 | 15:36 | 10:30 | Native | Invertebrates only | No Scavenging | N/A | 166 | 103 | 63 | 247 |
| EP2 | -35.7839 | 138.0639 | Lashmar Conservation Park | 06/11/2022 | 11/11/2022 | 15:36 | 10:30 | Native | Squamates only | No Scavenging | N/A | 150 | 25 | 125 | 30 |
| EP3 | -35.7994 | 138.0674 | Lashmar Conservation Park | 06/11/2022 | 11/11/2022 | 16:22 | 11:15 | Native | Accessible to all | Scavenging | Squamate | 123 | 0 | 123 | 0 |
| EP3 | -35.7994 | 138.0674 | Lashmar Conservation Park | 06/11/2022 | 11/11/2022 | 16:22 | 11:15 | Native | Birds only | Scavenging | Bird | 120 | 0 | 120 | 0 |
| EP3 | -35.7994 | 138.0674 | Lashmar Conservation Park | 06/11/2022 | 11/11/2022 | 16:22 | 11:15 | Native | Invertebrates only | No Scavenging | N/A | 153 | 36 | 117 | 272 |
| EP3 | -35.7994 | 138.0674 | Lashmar Conservation Park | 06/11/2022 | 11/11/2022 | 16:22 | 11:15 | Native | Squamates only | Scavenging | Squamate | 116 | 0 | 116 | 0 |
| FB1 | -35.1717 | 137.1669 | Foul Bay | 27/03/2022 | 01/04/2022 | 10:30 | 10:00 | Intermediate | Accessible to all | No Scavenging | N/A | 138 | 70 | 68 | 150 |
| FB1 | -35.1717 | 137.1669 | Foul Bay | 18/11/2022 | 23/11/2022 | 10:45 | 10:30 | Intermediate | Accessible to all | No Scavenging | N/A | 141 | 55 | 86 | 600 |
| FB1 | -35.1717 | 137.1669 | Foul Bay | 27/03/2022 | 01/04/2022 | 10:30 | 10:00 | Intermediate | Birds only | No Scavenging | N/A | 158 | 36 | 122 | 2 |
| FB1 | -35.1717 | 137.1669 | Foul Bay | 18/11/2022 | 23/11/2022 | 10:45 | 10:30 | Intermediate | Birds only | No Scavenging | N/A | 175 | 127 | 48 | 480 |
| FB1 | -35.1717 | 137.1669 | Foul Bay | 27/03/2022 | 01/04/2022 | 10:30 | 10:00 | Intermediate | Invertebrates only | No Scavenging | N/A | 150 | 26 | 124 | 1 |
| FB1 | -35.1717 | 137.1669 | Foul Bay | 18/11/2022 | 23/11/2022 | 10:45 | 10:30 | Intermediate | Invertebrates only | No Scavenging | N/A | 174 | 104 | 70 | 400 |
| FB1 | -35.1717 | 137.1669 | Foul Bay | 27/03/2022 | 01/04/2022 | 10:30 | 10:00 | Intermediate | Squamates only | No Scavenging | N/A | 136 | 37 | 99 | 690 |
| FB1 | -35.1717 | 137.1669 | Foul Bay | 18/11/2022 | 23/11/2022 | 10:45 | 10:30 | Intermediate | Squamates only | No Scavenging | N/A | 185 | 95 | 90 | 580 |
| FB2 | -35.176 | 137.1749 | Foul Bay | 27/03/2022 | 01/04/2022 | 11:00 | 10:30 | Intermediate | Accessible to all | No Scavenging | N/A | 160 | 49 | 111 | 630 |
| FB2 | -35.176 | 137.1749 | Foul Bay | 18/11/2022 | 23/11/2022 | 11:15 | 11:30 | Intermediate | Accessible to all | No Scavenging | N/A | 172 | 43 | 129 | 1044 |
| FB2 | -35.176 | 137.1749 | Foul Bay | 27/03/2022 | 01/04/2022 | 11:00 | 10:30 | Intermediate | Birds only | No Scavenging | N/A | 161 | 30 | 131 | 11 |
| FB2 | -35.176 | 137.1749 | Foul Bay | 18/11/2022 | 23/11/2022 | 11:15 | 11:30 | Intermediate | Birds only | No Scavenging | N/A | 152 | 125 | 27 | 230 |
| FB2 | -35.176 | 137.1749 | Foul Bay | 27/03/2022 | 01/04/2022 | 11:00 | 10:30 | Intermediate | Invertebrates only | No Scavenging | N/A | 129 | 47 | 82 | 410 |
| FB2 | -35.176 | 137.1749 | Foul Bay | 18/11/2022 | 23/11/2022 | 11:15 | 11:30 | Intermediate | Invertebrates only | No Scavenging | N/A | 158 | 78 | 80 | 670 |
| FB2 | -35.176 | 137.1749 | Foul Bay | 27/03/2022 | 01/04/2022 | 11:00 | 10:30 | Intermediate | Squamates only | No Scavenging | N/A | 135 | 65 | 70 | 640 |
| FB2 | -35.176 | 137.1749 | Foul Bay | 18/11/2022 | 23/11/2022 | 11:15 | 11:30 | Intermediate | Squamates only | No Scavenging | N/A | 172 | 52 | 120 | 760 |
| FB3 | -35.1811 | 137.185 | Foul Bay | 27/03/2022 | 01/04/2022 | 11:30 | 11:00 | Intermediate | Accessible to all | No Scavenging | N/A | 136 | 36 | 100 | 170 |
| FB3 | -35.1811 | 137.185 | Foul Bay | 18/11/2022 | 23/11/2022 | 12:00 | 12:30 | Intermediate | Accessible to all | No Scavenging | N/A | 192 | 140 | 52 | 440 |
| FB3 | -35.1811 | 137.185 | Foul Bay | 27/03/2022 | 01/04/2022 | 11:30 | 11:00 | Intermediate | Birds only | No Scavenging | N/A | 143 | 58 | 85 | 490 |
| FB3 | -35.1811 | 137.185 | Foul Bay | 18/11/2022 | 23/11/2022 | 12:00 | 12:30 | Intermediate | Birds only | No Scavenging | N/A | 192 | 180 | 12 | 200 |
| FB3 | -35.1811 | 137.185 | Foul Bay | 27/03/2022 | 01/04/2022 | 11:30 | 11:00 | Intermediate | Invertebrates only | No Scavenging | N/A | 154 | 50 | 104 | 840 |
| FB3 | -35.1811 | 137.185 | Foul Bay | 18/11/2022 | 23/11/2022 | 12:00 | 12:30 | Intermediate | Invertebrates only | No Scavenging | N/A | 173 | 130 | 43 | 300 |
| FB3 | -35.1811 | 137.185 | Foul Bay | 27/03/2022 | 01/04/2022 | 11:30 | 11:00 | Intermediate | Squamates only | No Scavenging | N/A | 140 | 36 | 104 | 2 |
| FB3 | -35.1811 | 137.185 | Foul Bay | 18/11/2022 | 23/11/2022 | 12:00 | 12:30 | Intermediate | Squamates only | No Scavenging | N/A | 188 | 113 | 75 | 450 |
| LB1 | -34.9133 | 137.1844 | Leven Beach | 24/03/2022 | 29/03/2022 | 10:00 | 10:30 | Disturbed | Accessible to all | Scavenging | Bird | 142 | 69 | 73 | 1 |
| LB1 | -34.9133 | 137.1844 | Leven Beach | 20/10/2022 | 25/10/2022 | 10:00 | 10:00 | Disturbed | Accessible to all | Scavenging | Bird | 169 | 0 | 169 | 0 |
| LB1 | -34.9133 | 137.1844 | Leven Beach | 24/03/2022 | 29/03/2022 | 10:00 | 10:30 | Disturbed | Birds only | Scavenging | Bird | 141 | 0 | 141 | 0 |
| LB1 | -34.9133 | 137.1844 | Leven Beach | 20/10/2022 | 25/10/2022 | 10:00 | 10:00 | Disturbed | Birds only | Scavenging | Bird | 172 | 0 | 172 | 0 |
| LB1 | -34.9133 | 137.1844 | Leven Beach | 24/03/2022 | 29/03/2022 | 10:00 | 10:30 | Disturbed | Invertebrates only | No Scavenging | N/A | 128 | 91 | 37 | 100 |
| LB1 | -34.9133 | 137.1844 | Leven Beach | 20/10/2022 | 25/10/2022 | 10:00 | 10:00 | Disturbed | Invertebrates only | No Scavenging | N/A | 183 | 57 | 126 | 797 |
| LB1 | -34.9133 | 137.1844 | Leven Beach | 24/03/2022 | 29/03/2022 | 10:00 | 10:30 | Disturbed | Squamates only | No Scavenging | N/A | 155 | 68 | 87 | 200 |
| LB1 | -34.9133 | 137.1844 | Leven Beach | 20/10/2022 | 25/10/2022 | 10:00 | 10:00 | Disturbed | Squamates only | No Scavenging | N/A | 175 | 56 | 119 | 610 |
| LB2 | -34.9134 | 137.2059 | Leven Beach | 24/03/2022 | 29/03/2022 | 10:30 | 11:30 | Disturbed | Accessible to all | Scavenging | Mammal | 141 | 43 | 98 | 6 |
| LB2 | -34.9134 | 137.2059 | Leven Beach | 20/10/2022 | 25/10/2022 | 10:45 | 11:15 | Disturbed | Accessible to all | Scavenging | Bird | 172 | 0 | 172 | 0 |
| LB2 | -34.9134 | 137.2059 | Leven Beach | 24/03/2022 | 29/03/2022 | 10:30 | 11:30 | Disturbed | Birds only | Scavenging | Bird | 131 | 32 | 99 | 3 |
| LB2 | -34.9134 | 137.2059 | Leven Beach | 20/10/2022 | 25/10/2022 | 10:45 | 11:15 | Disturbed | Birds only | Scavenging | Bird | 160 | 0 | 160 | 0 |
| LB2 | -34.9134 | 137.2059 | Leven Beach | 24/03/2022 | 29/03/2022 | 10:30 | 11:30 | Disturbed | Invertebrates only | No Scavenging | N/A | 125 | 81 | 44 | 18 |
| LB2 | -34.9134 | 137.2059 | Leven Beach | 20/10/2022 | 25/10/2022 | 10:45 | 11:15 | Disturbed | Invertebrates only | No Scavenging | N/A | 183 | 39 | 144 | 410 |
| LB2 | -34.9134 | 137.2059 | Leven Beach | 24/03/2022 | 29/03/2022 | 10:30 | 11:30 | Disturbed | Squamates only | No Scavenging | N/A | 137 | 80 | 57 | 23 |
| LB2 | -34.9134 | 137.2059 | Leven Beach | 20/10/2022 | 25/10/2022 | 10:45 | 11:15 | Disturbed | Squamates only | No Scavenging | N/A | 178 | 60 | 118 | 270 |
| LB3 | -34.9072 | 137.2264 | Leven Beach | 24/03/2022 | 29/03/2022 | 11:30 | 12:30 | Disturbed | Accessible to all | Scavenging | Mammal | 124 | 33 | 91 | 0 |
| LB3 | -34.9072 | 137.2264 | Leven Beach | 20/10/2022 | 25/10/2022 | 11:45 | 12:00 | Disturbed | Accessible to all | Scavenging | Bird | 136 | 0 | 136 | 0 |
| LB3 | -34.9072 | 137.2264 | Leven Beach | 24/03/2022 | 29/03/2022 | 11:30 | 12:30 | Disturbed | Birds only | No Scavenging | N/A | 134 | 83 | 51 | 100 |
| LB3 | -34.9072 | 137.2264 | Leven Beach | 20/10/2022 | 25/10/2022 | 11:45 | 12:00 | Disturbed | Birds only | Scavenging | Bird | 135 | 0 | 135 | 0 |
| LB3 | -34.9072 | 137.2264 | Leven Beach | 24/03/2022 | 29/03/2022 | 11:30 | 12:30 | Disturbed | Invertebrates only | No Scavenging | N/A | 129 | 69 | 60 | 1 |
| LB3 | -34.9072 | 137.2264 | Leven Beach | 20/10/2022 | 25/10/2022 | 11:45 | 12:00 | Disturbed | Invertebrates only | No Scavenging | N/A | 152 | 38 | 114 | 0 |
| LB3 | -34.9072 | 137.2264 | Leven Beach | 24/03/2022 | 29/03/2022 | 11:30 | 12:30 | Disturbed | Squamates only | No Scavenging | N/A | 148 | 53 | 95 | 7 |
| LB3 | -34.9072 | 137.2264 | Leven Beach | 20/10/2022 | 25/10/2022 | 11:45 | 12:00 | Disturbed | Squamates only | No Scavenging | N/A | 151 | 34 | 117 | 12 |
| PD1 | -35.1507 | 137.339 | Point Davenport | 07/04/2022 | 12/04/2022 | 10:30 | 11:30 | Disturbed | Accessible to all | Scavenging | Mammal | 61 | 13 | 48 | 0 |
| PD1 | -35.1507 | 137.339 | Point Davenport | 17/11/2022 | 22/11/2022 | 10:30 | 11:00 | Disturbed | Accessible to all | Scavenging | Bird | 138 | 0 | 138 | 0 |
| PD1 | -35.1507 | 137.339 | Point Davenport | 07/04/2022 | 12/04/2022 | 10:30 | 11:30 | Disturbed | Birds only | No Scavenging | N/A | 61 | 30 | 31 | 0 |
| PD1 | -35.1507 | 137.339 | Point Davenport | 17/11/2022 | 22/11/2022 | 10:30 | 11:00 | Disturbed | Birds only | No Scavenging | N/A | 165 | 15 | 150 | 1 |
| PD1 | -35.1507 | 137.339 | Point Davenport | 07/04/2022 | 12/04/2022 | 10:30 | 11:30 | Disturbed | Invertebrates only | No Scavenging | N/A | 66 | 40 | 26 | 0 |
| PD1 | -35.1507 | 137.339 | Point Davenport | 17/11/2022 | 22/11/2022 | 10:30 | 11:00 | Disturbed | Invertebrates only | No Scavenging | N/A | 143 | 45 | 98 | 909 |
| PD1 | -35.1507 | 137.339 | Point Davenport | 07/04/2022 | 12/04/2022 | 10:30 | 11:30 | Disturbed | Squamates only | No Scavenging | N/A | 56 | 25 | 31 | 110 |
| PD1 | -35.1507 | 137.339 | Point Davenport | 17/11/2022 | 22/11/2022 | 10:30 | 11:00 | Disturbed | Squamates only | Scavenging | Bird | 125 | 20 | 105 | 5 |
| PD2 | -35.1629 | 137.3352 | Point Davenport | 07/04/2022 | 12/04/2022 | 11:30 | 12:00 | Disturbed | Accessible to all | Scavenging | Mammal | 65 | 25 | 40 | 7 |
| PD2 | -35.1629 | 137.3352 | Point Davenport | 17/11/2022 | 22/11/2022 | 11:30 | 12:00 | Disturbed | Accessible to all | No Scavenging | N/A | 128 | 30 | 98 | 508 |
| PD2 | -35.1629 | 137.3352 | Point Davenport | 07/04/2022 | 12/04/2022 | 11:30 | 12:00 | Disturbed | Birds only | No Scavenging | N/A | 62 | 32 | 30 | 13 |
| PD2 | -35.1629 | 137.3352 | Point Davenport | 17/11/2022 | 22/11/2022 | 11:30 | 12:00 | Disturbed | Birds only | No Scavenging | N/A | 169 | 140 | 29 | 412 |
| PD2 | -35.1629 | 137.3352 | Point Davenport | 07/04/2022 | 12/04/2022 | 11:30 | 12:00 | Disturbed | Invertebrates only | No Scavenging | N/A | 60 | 19 | 41 | 0 |
| PD2 | -35.1629 | 137.3352 | Point Davenport | 17/11/2022 | 22/11/2022 | 11:30 | 12:00 | Disturbed | Invertebrates only | No Scavenging | N/A | 123 | 25 | 98 | 1106 |
| PD2 | -35.1629 | 137.3352 | Point Davenport | 07/04/2022 | 12/04/2022 | 11:30 | 12:00 | Disturbed | Squamates only | No Scavenging | N/A | 61 | 35 | 26 | 0 |
| PD2 | -35.1629 | 137.3352 | Point Davenport | 17/11/2022 | 22/11/2022 | 11:30 | 12:00 | Disturbed | Squamates only | No Scavenging | N/A | 147 | 50 | 97 | 839 |
| PD3 | -35.1549 | 137.3331 | Point Davenport | 07/04/2022 | 12/04/2022 | 12:15 | 13:00 | Disturbed | Accessible to all | No Scavenging | N/A | 70 | 25 | 45 | 17 |
| PD3 | -35.1549 | 137.3331 | Point Davenport | 17/11/2022 | 22/11/2022 | 12:30 | 13:00 | Disturbed | Accessible to all | Scavenging | Bird | 143 | 0 | 143 | 0 |
| PD3 | -35.1549 | 137.3331 | Point Davenport | 07/04/2022 | 12/04/2022 | 12:15 | 13:00 | Disturbed | Birds only | No Scavenging | N/A | 61 | 38 | 23 | 40 |
| PD3 | -35.1549 | 137.3331 | Point Davenport | 17/11/2022 | 22/11/2022 | 12:30 | 13:00 | Disturbed | Birds only | Scavenging | Bird | 141 | 0 | 141 | 0 |
| PD3 | -35.1549 | 137.3331 | Point Davenport | 07/04/2022 | 12/04/2022 | 12:15 | 13:00 | Disturbed | Invertebrates only | No Scavenging | N/A | 70 | 21 | 49 | 0 |
| PD3 | -35.1549 | 137.3331 | Point Davenport | 17/11/2022 | 22/11/2022 | 12:30 | 13:00 | Disturbed | Invertebrates only | No Scavenging | N/A | 122 | 26 | 96 | 1153 |
| PD3 | -35.1549 | 137.3331 | Point Davenport | 07/04/2022 | 12/04/2022 | 12:15 | 13:00 | Disturbed | Squamates only | No Scavenging | N/A | 60 | 22 | 38 | 3 |
| PD3 | -35.1549 | 137.3331 | Point Davenport | 17/11/2022 | 22/11/2022 | 12:30 | 13:00 | Disturbed | Squamates only | No Scavenging | N/A | 164 | 25 | 139 | 12 |
| SCP1 | -35.8537 | 137.9701 | Simpson Conservation Park | 05/11/2022 | 10/11/2022 | 10:30 | 09:30 | Native | Accessible to all | Scavenging | Bird | 164 | 0 | 164 | 0 |
| SCP1 | -35.8537 | 137.9701 | Simpson Conservation Park | 05/11/2022 | 10/11/2022 | 10:30 | 09:30 | Native | Birds only | No Scavenging | N/A | 125 | 29 | 96 | 1133 |
| SCP1 | -35.8537 | 137.9701 | Simpson Conservation Park | 05/11/2022 | 10/11/2022 | 10:30 | 09:30 | Native | Invertebrates only | No Scavenging | N/A | 162 | 38 | 124 | 20 |
| SCP1 | -35.8537 | 137.9701 | Simpson Conservation Park | 05/11/2022 | 10/11/2022 | 10:30 | 09:30 | Native | Squamates only | Scavenging | Squamate | 143 | 0 | 143 | 0 |
| SCP2 | -35.856 | 137.9793 | Simpson Conservation Park | 05/11/2022 | 10/11/2022 | 11:20 | 10:20 | Native | Accessible to all | Scavenging | Bird | 166 | 0 | 166 | 0 |
| SCP2 | -35.856 | 137.9793 | Simpson Conservation Park | 05/11/2022 | 10/11/2022 | 11:20 | 10:20 | Native | Birds only | Scavenging | Squamate | 118 | 0 | 118 | 0 |
| SCP2 | -35.856 | 137.9793 | Simpson Conservation Park | 05/11/2022 | 10/11/2022 | 11:20 | 10:20 | Native | Invertebrates only | No Scavenging | N/A | 159 | 68 | 91 | 435 |
| SCP2 | -35.856 | 137.9793 | Simpson Conservation Park | 05/11/2022 | 10/11/2022 | 11:20 | 10:20 | Native | Squamates only | Scavenging | Squamate | 151 | 0 | 151 | 0 |
| SCP3 | -35.867 | 137.993 | Simpson Conservation Park | 05/11/2022 | 10/11/2022 | 12:30 | 11:10 | Native | Accessible to all | Scavenging | Squamate | 152 | 0 | 152 | 0 |
| SCP3 | -35.867 | 137.993 | Simpson Conservation Park | 05/11/2022 | 10/11/2022 | 12:30 | 11:10 | Native | Birds only | Scavenging | Bird | 144 | 0 | 144 | 0 |
| SCP3 | -35.867 | 137.993 | Simpson Conservation Park | 05/11/2022 | 10/11/2022 | 12:30 | 11:10 | Native | Invertebrates only | No Scavenging | N/A | 140 | 36 | 104 | 748 |
| SCP3 | -35.867 | 137.993 | Simpson Conservation Park | 05/11/2022 | 10/11/2022 | 12:30 | 11:10 | Native | Squamates only | No Scavenging | N/A | 125 | 33 | 92 | 60 |
| WB1 | -35.1199 | 137.0878 | Warrenben | 25/03/2022 | 30/03/2022 | 10:30 | 09:30 | Intermediate | Accessible to all | Scavenging | Bird | 124 | 0 | 124 | 0 |
| WB1 | -35.1199 | 137.0878 | Warrenben | 22/10/2022 | 27/10/2022 | 10:15 | 09:50 | Intermediate | Accessible to all | Scavenging | Bird | 153 | 0 | 153 | 0 |
| WB1 | -35.1199 | 137.0878 | Warrenben | 25/03/2022 | 30/03/2022 | 10:30 | 09:30 | Intermediate | Birds only | Scavenging | Bird | 151 | 0 | 151 | 0 |
| WB1 | -35.1199 | 137.0878 | Warrenben | 22/10/2022 | 27/10/2022 | 10:15 | 09:50 | Intermediate | Birds only | Scavenging | Bird | 127 | 0 | 127 | 0 |
| WB1 | -35.1199 | 137.0878 | Warrenben | 25/03/2022 | 30/03/2022 | 10:30 | 09:30 | Intermediate | Invertebrates only | No Scavenging | N/A | 136 | 75 | 61 | 81 |
| WB1 | -35.1199 | 137.0878 | Warrenben | 22/10/2022 | 27/10/2022 | 10:15 | 09:50 | Intermediate | Invertebrates only | No Scavenging | N/A | 153 | 60 | 93 | 850 |
| WB1 | -35.1199 | 137.0878 | Warrenben | 25/03/2022 | 30/03/2022 | 10:30 | 09:30 | Intermediate | Squamates only | Scavenging | Squamate | 155 | 0 | 155 | 0 |
| WB1 | -35.1199 | 137.0878 | Warrenben | 22/10/2022 | 27/10/2022 | 10:15 | 09:50 | Intermediate | Squamates only | Scavenging | Squamate | 138 | 0 | 138 | 0 |
| WB2 | -35.1083 | 137.0418 | Warrenben | 25/03/2022 | 30/03/2022 | 11:30 | 10:30 | Intermediate | Accessible to all | Scavenging | Squamate | 157 | 0 | 157 | 0 |
| WB2 | -35.1083 | 137.0418 | Warrenben | 22/10/2022 | 27/10/2022 | 11:00 | 10:45 | Intermediate | Accessible to all | Scavenging | Squamate | 166 | 0 | 166 | 0 |
| WB2 | -35.1083 | 137.0418 | Warrenben | 25/03/2022 | 30/03/2022 | 11:30 | 10:30 | Intermediate | Birds only | No Scavenging | N/A | 127 | 89 | 38 | 2 |
| WB2 | -35.1083 | 137.0418 | Warrenben | 22/10/2022 | 27/10/2022 | 11:00 | 10:45 | Intermediate | Birds only | Scavenging | Bird | 142 | 0 | 142 | 0 |
| WB2 | -35.1083 | 137.0418 | Warrenben | 25/03/2022 | 30/03/2022 | 11:30 | 10:30 | Intermediate | Invertebrates only | No Scavenging | N/A | 160 | 36 | 124 | 1 |
| WB2 | -35.1083 | 137.0418 | Warrenben | 22/10/2022 | 27/10/2022 | 11:00 | 10:45 | Intermediate | Invertebrates only | No Scavenging | N/A | 125 | 24 | 101 | 0 |
| WB2 | -35.1083 | 137.0418 | Warrenben | 25/03/2022 | 30/03/2022 | 11:30 | 10:30 | Intermediate | Squamates only | No Scavenging | N/A | 143 | 76 | 67 | 40 |
| WB2 | -35.1083 | 137.0418 | Warrenben | 22/10/2022 | 27/10/2022 | 11:00 | 10:45 | Intermediate | Squamates only | Scavenging | Squamate | 158 | 0 | 158 | 0 |
| WB3 | -35.1497 | 137.0363 | Warrenben | 25/03/2022 | 30/03/2022 | 13:00 | 11:30 | Intermediate | Accessible to all | No Scavenging | N/A | 124 | 55 | 69 | 320 |
| WB3 | -35.1497 | 137.0363 | Warrenben | 22/10/2022 | 27/10/2022 | 12:25 | 12:00 | Intermediate | Accessible to all | No Scavenging | N/A | 160 | 27 | 133 | 0 |
| WB3 | -35.1497 | 137.0363 | Warrenben | 25/03/2022 | 30/03/2022 | 13:00 | 11:30 | Intermediate | Birds only | No Scavenging | N/A | 136 | 65 | 71 | 210 |
| WB3 | -35.1497 | 137.0363 | Warrenben | 22/10/2022 | 27/10/2022 | 12:25 | 12:00 | Intermediate | Birds only | No Scavenging | N/A | 132 | 45 | 87 | 680 |
| WB3 | -35.1497 | 137.0363 | Warrenben | 25/03/2022 | 30/03/2022 | 13:00 | 11:30 | Intermediate | Invertebrates only | No Scavenging | N/A | 143 | 104 | 39 | 1 |
| WB3 | -35.1497 | 137.0363 | Warrenben | 22/10/2022 | 27/10/2022 | 12:25 | 12:00 | Intermediate | Invertebrates only | No Scavenging | N/A | 135 | 18 | 117 | 2 |
| WB3 | -35.1497 | 137.0363 | Warrenben | 25/03/2022 | 30/03/2022 | 13:00 | 11:30 | Intermediate | Squamates only | No Scavenging | N/A | 148 | 18 | 130 | 0 |
| WB3 | -35.1497 | 137.0363 | Warrenben | 22/10/2022 | 27/10/2022 | 12:25 | 12:00 | Intermediate | Squamates only | No Scavenging | N/A | 133 | 17 | 116 | 3 |
| YH1 | -35.0367 | 137.2057 | Yorke Highway | 26/03/2022 | 31/03/2022 | 10:30 | 10:00 | Disturbed | Accessible to all | Scavenging | Bird | 145 | 41 | 104 | 0 |
| YH1 | -35.0367 | 137.2057 | Yorke Highway | 21/10/2022 | 26/10/2022 | 08:50 | 10:10 | Disturbed | Accessible to all | Scavenging | Bird | 165 | 18 | 147 | 10 |
| YH1 | -35.0367 | 137.2057 | Yorke Highway | 26/03/2022 | 31/03/2022 | 10:30 | 10:00 | Disturbed | Birds only | No Scavenging | N/A | 149 | 75 | 74 | 50 |
| YH1 | -35.0367 | 137.2057 | Yorke Highway | 21/10/2022 | 26/10/2022 | 08:50 | 10:10 | Disturbed | Birds only | Scavenging | Bird | 131 | 0 | 131 | 0 |
| YH1 | -35.0367 | 137.2057 | Yorke Highway | 26/03/2022 | 31/03/2022 | 10:30 | 10:00 | Disturbed | Invertebrates only | No Scavenging | N/A | 152 | 17 | 135 | 0 |
| YH1 | -35.0367 | 137.2057 | Yorke Highway | 21/10/2022 | 26/10/2022 | 08:50 | 10:10 | Disturbed | Invertebrates only | No Scavenging | N/A | 138 | 22 | 116 | 32 |
| YH1 | -35.0367 | 137.2057 | Yorke Highway | 26/03/2022 | 31/03/2022 | 10:30 | 10:00 | Disturbed | Squamates only | No Scavenging | N/A | 130 | 8 | 122 | 0 |
| YH1 | -35.0367 | 137.2057 | Yorke Highway | 21/10/2022 | 26/10/2022 | 08:50 | 10:10 | Disturbed | Squamates only | No Scavenging | N/A | 133 | 21 | 112 | 0 |
| YH2 | -35.0507 | 137.2148 | Yorke Highway | 26/03/2022 | 31/03/2022 | 11:30 | 11:00 | Disturbed | Accessible to all | No Scavenging | N/A | 158 | 87 | 71 | 0 |
| YH2 | -35.0507 | 137.2148 | Yorke Highway | 21/10/2022 | 26/10/2022 | 09:25 | 11:00 | Disturbed | Accessible to all | No Scavenging | N/A | 124 | 20 | 104 | 227 |
| YH2 | -35.0507 | 137.2148 | Yorke Highway | 26/03/2022 | 31/03/2022 | 11:30 | 11:00 | Disturbed | Birds only | No Scavenging | N/A | 150 | 97 | 53 | 1 |
| YH2 | -35.0507 | 137.2148 | Yorke Highway | 21/10/2022 | 26/10/2022 | 09:25 | 11:00 | Disturbed | Birds only | Scavenging | Bird | 143 | 27 | 116 | 120 |
| YH2 | -35.0507 | 137.2148 | Yorke Highway | 26/03/2022 | 31/03/2022 | 11:30 | 11:00 | Disturbed | Invertebrates only | No Scavenging | N/A | 156 | 86 | 70 | 10 |
| YH2 | -35.0507 | 137.2148 | Yorke Highway | 21/10/2022 | 26/10/2022 | 09:25 | 11:00 | Disturbed | Invertebrates only | No Scavenging | N/A | 138 | 27 | 111 | 121 |
| YH2 | -35.0507 | 137.2148 | Yorke Highway | 26/03/2022 | 31/03/2022 | 11:30 | 11:00 | Disturbed | Squamates only | No Scavenging | N/A | 139 | 37 | 102 | 0 |
| YH2 | -35.0507 | 137.2148 | Yorke Highway | 21/10/2022 | 26/10/2022 | 09:25 | 11:00 | Disturbed | Squamates only | No Scavenging | N/A | 170 | 30 | 140 | 12 |
| YH3 | -35.0388 | 137.2141 | Yorke Highway | 26/03/2022 | 31/03/2022 | 12:30 | 11:30 | Disturbed | Accessible to all | No Scavenging | N/A | 143 | 53 | 90 | 590 |
| YH3 | -35.0388 | 137.2141 | Yorke Highway | 21/10/2022 | 26/10/2022 | 10:00 | 12:00 | Disturbed | Accessible to all | Scavenging | Bird | 151 | 0 | 151 | 0 |
| YH3 | -35.0388 | 137.2141 | Yorke Highway | 26/03/2022 | 31/03/2022 | 12:30 | 11:30 | Disturbed | Birds only | No Scavenging | N/A | 141 | 81 | 60 | 430 |
| YH3 | -35.0388 | 137.2141 | Yorke Highway | 21/10/2022 | 26/10/2022 | 10:00 | 12:00 | Disturbed | Birds only | Scavenging | Bird | 129 | 34 | 95 | 142 |
| YH3 | -35.0388 | 137.2141 | Yorke Highway | 26/03/2022 | 31/03/2022 | 12:30 | 11:30 | Disturbed | Invertebrates only | No Scavenging | N/A | 158 | 60 | 98 | 710 |
| YH3 | -35.0388 | 137.2141 | Yorke Highway | 21/10/2022 | 26/10/2022 | 10:00 | 12:00 | Disturbed | Invertebrates only | No Scavenging | N/A | 137 | 22 | 115 | 24 |
| YH3 | -35.0388 | 137.2141 | Yorke Highway | 26/03/2022 | 31/03/2022 | 12:30 | 11:30 | Disturbed | Squamates only | No Scavenging | N/A | 135 | 22 | 113 | 0 |
| YH3 | -35.0388 | 137.2141 | Yorke Highway | 21/10/2022 | 26/10/2022 | 10:00 | 12:00 | Disturbed | Squamates only | No Scavenging | N/A | 135 | 20 | 115 | 90 |


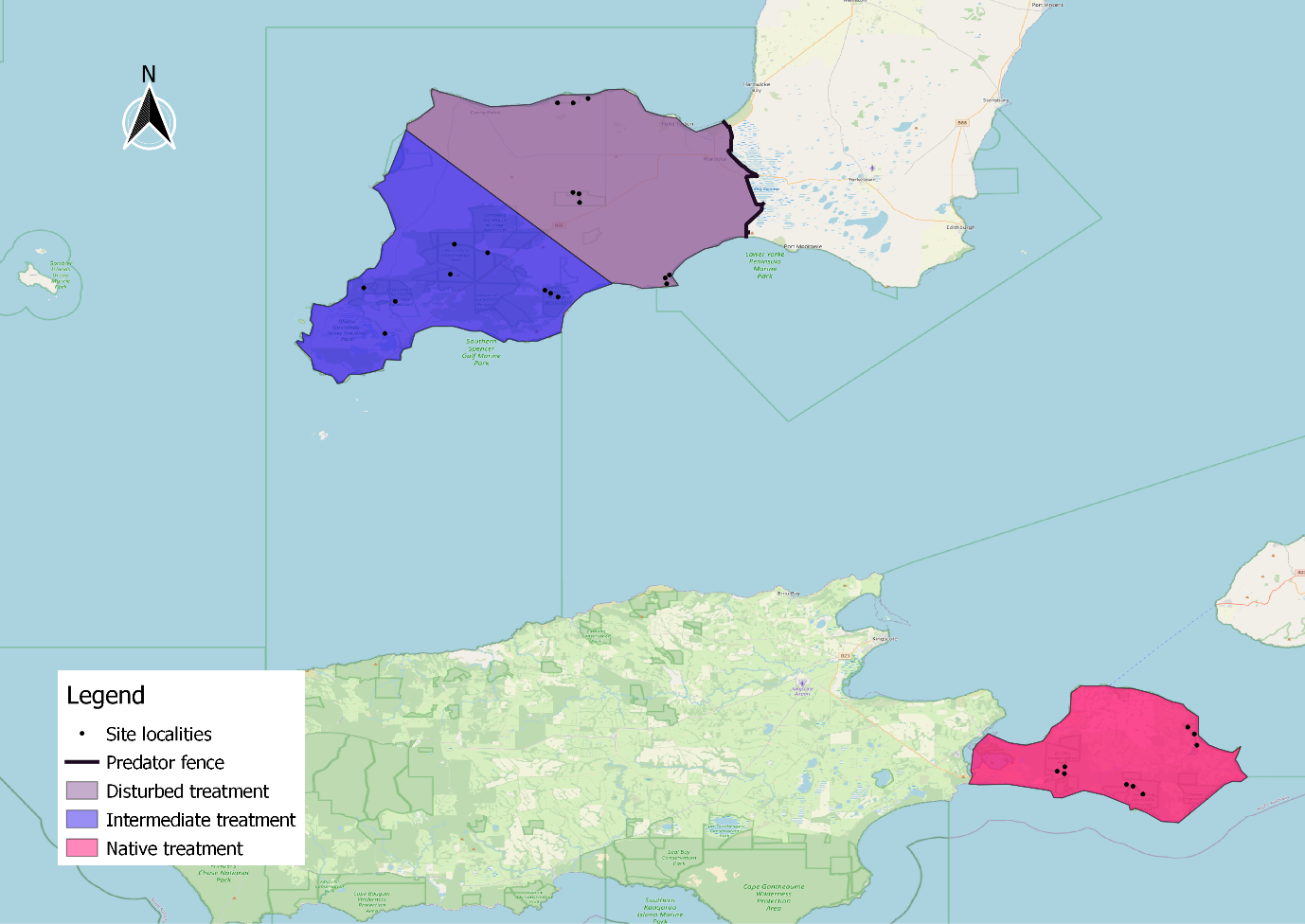


**Figure S1.** The study area with each scavenger community treatment highlighted, with site localities shown.
